# On-Line Thermally Induced Evolved Gas Analysis: An Update—Part 1: EGA-MS

**DOI:** 10.3390/molecules27113518

**Published:** 2022-05-30

**Authors:** Roberta Risoluti, Giuseppina Gullifa, Laura Barone, Elena Papa, Stefano Materazzi

**Affiliations:** Department of Chemistry, “Sapienza” Università di Roma, P.le A.Moro 5, 00185 Rome, Italy; roberta.risoluti@uniroma1.it (R.R.); giuseppina.gullifa@uniroma1.it (G.G.); laura.barone@uniroma1.it (L.B.); elena.papa@uniroma1.it (E.P.)

**Keywords:** EGA, evolved gas analysis, MS, mass spectrometry, EGA-MS, TG-MS

## Abstract

Advances in on-line thermally induced evolved gas analysis (OLTI-EGA) have been systematically reported by our group to update their applications in several different fields and to provide useful starting references. The importance of an accurate interpretation of the thermally-induced reaction mechanism which involves the formation of gaseous species is necessary to obtain the characterization of the evolved products. In this review, applications of Evolved Gas Analysis (EGA) performed by on-line coupling heating devices to mass spectrometry (EGA-MS), are reported. Reported references clearly demonstrate that the characterization of the nature of volatile products released by a substance subjected to a controlled temperature program allows us to prove a supposed reaction or composition, either under isothermal or under heating conditions. Selected 2019, 2020, and 2021 references are collected and briefly described in this review.

## 1. Introduction

On-Line Thermally Induced Evolved Gas Analysis (OLTI-EGA) includes several hyphenated techniques that provide useful information on the gaseous products released when thermally induced reactions take place.

Being able to obtain the EGA allows one to correctly interpret the releasing process or the decomposition mechanism. Instead of pyrolizers, thermoanalytical techniques are the most frequent instruments coupled to mass spectrometers by means of a heated transfer line, since at the same time both characterization and quantification of each single gaseous evolution process are obtained.

Since 1997, periodic reviews have been proposed by our group to report selected advances in EGA techniques, showing applications obtained by coupling mass spectrometry (EGA-MS) [1,2,3,4,5,6] or infrared spectroscopy (EGA-IR) [7,8,9,10,11,12].

A recent review on EGA-MS with Soft Photoionization for the chemical description of petroleum, petroleum-derived materials, and alternative feedstocks was also reported by Ruger and coworkers [13].

Very recent analytical applications of evolved gas analysis, selected from those published in 2019, 2020, and 2021, are collected in this review and briefly described, for the purpose of establishing a useful starting reference. The number of publications on hyphenated techniques continues to grow in areas of specialized applications; as a consequence, it is not unusual for an article on the topic to appear in an unfamiliar journal or a trade-specific publication. This review aims to help researchers easily find applications that are sometimes difficult to locate.

## 2. Applications to Polymers

The synthesis, characterization, and thermal and fungal behavior of a green thermoset based on epoxidized soybean oil, castor oil maleic anhydride adduct, and methyl nadic anhydride was reported by Mustata and coworkers. By varying the crosslinking agent ratio, resins with different degrees of stiffness can be obtained, and are suitable for green thermoset coatings. Simultaneous EGA by FT-IR and MS coupling was used to establish the crosslinking behavior, to characterize the evolved gases, and to describe the thermal decomposition mechanisms associated with their corresponding kinetic parameters. The main identified gaseous fragments were water, carbon dioxide, saturated and unsaturated hydrocarbons, and carboxylic derivatives [14].

The thermal and thermo-oxidative stability of some aromatic poly(aryl ether ether ketone)s as well as the degradation mechanism were studied by the simultaneous MS/FTIR evolved gas analysis system in both air and helium. It was demonstrated that the degradation mechanism of the analyzed samples is clearly influenced by their structure and working atmosphere [15].

Ethylene vinyl acetate is known to deteriorate on prolonged exposure to sunlight. Yamada and coworkers reported for the first time the in situ identification of gases liberated during UV irradiation, such as H_2_O, CO_2_, ketones, acetic acid, and lactones. In addition, the deterioration of the thermal stability on aging was confirmed using EGA-MS. Findings were useful for a superior understanding of the breakdown, and lead to the development of UV-resistant encapsulating materials [16].

A big challenge for civilization in energy saving/waste management can be “the regeneration of monomers from the waste plastics followed by their re-polymerization” using an ideal recycling method. Godiya et al. provided an effective and practical prototype for the recycling of waste PMMA scraps and thus a reduction in pollution caused by the landfilling of waste PMMA scraps, by investigating the thermal depolymerization of poly-(methyl methacrylate) using several techniques, including EGA-MS. In this process, the polymer chains were decomposed to methyl methacrylate in high yield and the degradation species were thoroughly characterized. Results showed that the obtained methyl methacrylate contains traces of byproducts, nonpolymerizable, and their presence either interrupts the polymerization reaction or reduces the quality of re-polymerized PMMA [17]. Co-pyrolysis of biomass and waste plastics is the preferred technology for enhancing the production of fuels and valuable chemicals. Ma et al. studied the co-pyrolysis of milled wood lignin with polyethylene and polypropylene at various weight ratios. Evolved gas analysis–mass spectrometry and product recovery tests using a fixed-bed reactor were applied to investigate the physical prevention of melted polymers on the interactions between the vapored intermediates [18].

Light is a determining factor in the discoloration of plastics, and photodegradation processes can affect the molecular structures of both the polymer and colorants. Mass spectrometry techniques for the characterization of evolved gas analysis were successfully employed for characterizing the plastic formulations and degradation. The identification of phthalic compounds in aged β-naphthol powders opens new avenues for studies on their degradation. These experimental approaches and analytical methods in studying the discoloration of historical plastics are novel, proving their efficacy, reliability, and potentiality [19].

Direct analysis in real-time mass spectrometry (DART-MS) provides qualitative information about additives and polymer composition. Cody and coworkers heated, on disposable copper stages, several industrial polymers, and the evolved gases were introduced directly into a DART ion source through a glass tee. Time- and temperature-dependent mass spectra were acquired using a high-resolution time-of-flight mass spectrometer. Positive-ion DART mass spectra exhibited peak series differing by monomer masses, often accompanied by a peak corresponding to the protonated monomer. Thermal desorption provided characteristic temperature profiles for volatile species such as polymer additives and polymer pyrolysis products [20].

Over the last few decades, bio-based polymers have attracted considerable attention from both academic and industrial fields regarding the minimization of the environmental impact arising from the excessive use of petrochemically-based polymeric materials. PEV (poly-ethylene vanillate), a monomer originating from lignin-derived vanillic acid, has shown promising thermal and mechanical properties. The effects of three different catalysts on the synthesis of PEV via a two-stage melt polycondensation method were investigated by Xanthopoulou et al. The progress of the reaction was assessed using various complementary techniques, including evolved gas analysis by mass spectrometry [21].

Chain-end functionalized polybutadiene polymers have a widespread application in composite solid propellants. The curing of these polymers is affected by the reactions at the terminal groups with isocyanates or aziridines if the functional groups are hydroxyl or carboxyl, respectively. However, the high toxicity of isocyanates and aziridines demands alternate cure methods. A paper by Sasidharakurup et al. presented the elucidation of the mechanism. The decomposition aspects of the cured triazoline system were evaluated by EGA-MS, with the demonstration that the decomposition reaction yields more volatile products like nitrogen, carbon dioxide, 1,4 butadiene, and 4-vinylcyclohexene, conducive for propellant applications [22].

Based on the cross-linking reaction of bisphenol A epoxy resin and liquid carboxy-terminated poly(tetrafluoroethylene-hexafluoropropylene-vinylidene fluoride) copolymer, an elastomer containing fluoroolefin segment was prepared by Tang and coworkers. Coupled MS-FTIR evolved gas analysis was used to detect the thermal decomposition products and to discuss the thermal decomposition mechanisms [23].

Quantitative analysis of red phosphorus in polypropylene was studied using a temperature programmable pyrolyzer in combination with a mass spectrometer. Evolved gas analysis profiles were obtained by continuous measurements of evolved gases from a sample while heating the sample at a constant heating rate. During heating of the sample, red phosphorus sublimates into P4 molecules, which have characteristic ions (*m*/*z* 31, 62, 93, and 124). Red phosphorus in polypropylene was determined from the *m*/*z* 62 ion peak area of the EGA profile with good reproducibility [24].

## 3. Applications to Complexes and Compounds

An overall decomposition mechanism of ammonium dinitramide in the gas phase functional theory (DFT) calculations was studied by Wang et al. A TG-MS system was used to verify the calculation results. In the thermal decomposition, gaseous ammonium dinitramide first decomposed into dinitraminic acid and NH_3_, followed by decomposition into other products with small molecules. Produced HNO_3_ and NH_3_ first combined to form NH_4_NO_3_. Both calculated and experimental results indicated that the product N_2_O is first formed in the thermal decomposition of ammonium dinitramide [25].

A new cyclo-pentazolate anion-based energetic cocrystal was synthesized by Xu and coworkers. The structure, thermal behavior, sensitivity, and detonation properties of NH_4_N_5_·1/6NH_4_Cl were studied. DSC-TG-MS combined techniques showed that the cocrystal has better thermal stability than NH_4_N_5_, exhibits low impact and friction sensitivity, and largely reduces the mechanical sensitivity [26].

The biobased protic ionic liquid crystal bis(2-hydroxyethyl) ammonium palmitate has been studied by Aviles et al. as a neat lubricant under linear reciprocating sliding at 75 °C, in the liquid crystalline region, and at 110 °C, above its melting point. The resulting characterization was also related to water content, as determined by EGA-MS [27].

Vapor-phase ortho-methylation of 4-chlorophenol with methanol was studied over a Mn_2_O_3_ catalyst with two kinds of morphologies. EGA-MS and other characterizations made it clear that methanol reduced 4-chlorophenol and its methide, which were the main side reactions [28].

In vitro cytotoxic evaluation, formation constants, and thermal stability of four mixed-ligand Co(II) complexes with *N*,*N*′,*N*″,*N*‴-tetrakis(2-pyridylmethyl)-1,4,8,11-tetraazacyclotetradecane were reported by Nicolic and coauthors. The thermal behavior of the complexes was investigated by simultaneous TG/DSC and EGA-MS measurements [29]. Studies over several years on imidazole derivative ligands suggested two main characteristic complex structures that are independent of the central metal ion. By the thermally induced decomposition behaviors, two different systematic decomposition trends were proposed. The final goal of these serial studies has been to provide, by experimental evidence, a prediction model of thermal stability and typical decomposition behavior by comparing the structural characteristics of precipitated complexes [30]. Papadopoulos and coworkers investigated neutral Co(II) complexes with 2,2′-dipyridylamine and salicylaldehydes [31]. Franguelli et al. reported the characterization of di[carbonatotetraamminecobalt(III)] sulfate trihydrate compounds and the nature of its thermal decomposition products [32].

The thermal study, identification of intermediate solid products, evolved gas analysis during pyrolysis and oxidative decomposition of sodium complex of quercetin-5′-sulfonic acid were described by Maciolek and coworkers [33].

As a result of a ten-year-long study, Risoluti and Materazzi compared the information obtained by simple thermoanalytical characterizations of biomimetic complexes of imidazole substituted ligands with transition metal ions [34,35,36,37,38,39,40,41,42] with those completed by EGA-MS characterization [43,44,45,46,47,48,49,50,51,52,53,54,55,56] and demonstrated how fundamental an evolved gas analysis approach is to rigorously propose a typical thermal behavior.

Four new ligands and four new copper (II) coordination compounds were prepared and characterized by chemical, elemental analysis, cytotoxicity, and FTIR spectroscopy. The thermal properties of complexes in the solid-state were studied using thermogravimetric analysis coupled with mass spectrometry (TG-MS) under a dynamic flowing air atmosphere to analyze the principal volatile thermal decomposition and fragmentation products that evolved during thermolysis [57].

Tian et al. studied the thermal stability and the thermal decomposition mechanism of 1-((cyano-1-methylethyl) azo) formamide, an initiator that has the inherent property of being decomposed at high temperatures with considerable amounts of heat generated. The thermal hazard of CABN is the main consideration in the occurrence of serious accidents [58].

The ammonia absorption process of zirconium phosphate has been studied using the concentration-composition-isotherm X-ray diffraction and thermogravimetry-mass spectrometry. It was clarified that the equilibrium plateau concentration appeared due to two-phase coexistence [59].

Two novel mixed ligand complexes with the general formula [M_2_(4,4′-bpy)_1.5_(CBr_2_HCOO)_6_(H_2_O)_2_]_n_ (where 4,4′-bpy = 4,4′-bipyridine) were synthesized by Czylkowska et al. Thermal analysis was used to describe solid intermediate and final products of thermolysis. The coupling to a mass spectrometer was used to monitor principal volatile fragments that evolved during pyrolysis [60].

The purification of natural powder quartz was reported by Du and coworkers and was based on an integrated approach involving a reductive roasting pretreatment and acid leaching technology in the presence of ammonium sulphate. EGA-MS, color responses of quartz, and DFT were used to investigate the activation mechanism [61].

Jacobson et al. equilibrated carbon dioxide with molten sodium metasilicates and analyzed the results with postexposure carbon analyses and evolved gas analysis. EGA suggested two forms of CO_2_ solubility: dissolved as a molecule to a limited extent or primarily bound as a compound [62].

Among important environmentally-friendly organic compounds, dimethyl carbonate is becoming of interest and the need for its efficient synthesis is driving new catalysts and their cost-effective preparation. A series of KAlO_2_/γ-Al_2_O_3_ heterogeneous catalysts were prepared and characterized by spectroscopic techniques, TG-MS, ICP-OES, BET, and Hammett acid-base titration and tested for the synthesis via transesterification from ethylene carbonate with methanol. EGA was especially helpful during the calcination process [63]. Ionic liquids were investigated to provide relevant information on the thermal behavior [64].

The EGA thermal behavior of Au complexes was fundamental to proving that at first, a decarboxylation process occurs, as evidenced by the detection of CO_2_ [65].

A solid-phase guest exchange in anhydrous inclusion compounds with organic guests was used by Gatiatulin and coworkers to activate the inclusion properties without the presence of water. The initial inclusion compounds and the products of this exchange process were characterized using mass spectrometric analysis of evolved vapors and powder X-ray diffraction [66].

The effect of La content and its incorporation route on physicochemical properties of ZnO/Zn(Al,La)_2_O_4_ or mixed oxides with a spinel structure obtained from ZnAlLa layered double hydroxides was investigated by Antoniak-Jurak et al. Evolved gas analysis helped to reveal that introduction of lanthanum oxide over 2.4–2.8 wt% induces the phase separation of the ZnAl_2_O_4_ spinel, forming ZnO [67].

The fiber-like bis-(dimethylglyoximato) nickel(II) complex was successfully synthesized and characterized by Yao and coworkers using EGA by DSC-FTIR-MS coupled technique to describe the decomposition behavior and evolved gases [68].

Synthesis, crystal structure, and thermal behavior of magnesium 5,5′-bistetrazole-1,1′-diolate hexahydrate complexes were reported by Xiaojun and coworkers [69] while trivalent lanthanides (Eu-Ho) complexes with the valsartan ligand were studied by Ekawa et al. [70].

Several evolved gas analyses were performed to characterize Michael addition oligomers of acrylic acid by Fujita et al. to gain a better understanding of the reaction mechanisms during a runaway polymerization [71].

## 4. Applications to Metal-Organic Frameworks

The metal-organic frameworks (MOFs), as emerging materials, have attracted worldwide attention. In this view, a novel strategy of blending classic MOFs material UiO-66-NH_2_ to efficiently enhance the thermal stability and thermal aging resistance of silicon rubber was proposed by Xu et al. The possible heat resistance mechanism was explored by processing torque and EGA-MS characterizations, whose results suggested that even very low addition of UiO-66-NH_2_ could efficiently inhibit degradation of silicon rubber chains and obstruct heat transfer due to its low thermal conductivity [72].

The pyrolysis process of metal-organic frameworks is important for the study of the synthesis of MOFs-based carbon materials with high adsorption performance. The process can be successfully studied by EGA-MS to detect the gas products of the pyrolysis process. The main gas products in the pyrolysis of the solvothermal method obtained MOF were characterized and resulted in CO, CO_2,_ and benzene [73].

## 5. Applications to Catalyst

Transition metal phosphides are promising materials for catalysis and their synthesis procedures commonly require costly or hazardous reagents. Tong and coworkers adopted a yeast-extracted nucleic acid as an environmentally benign non-metal source to develop bifunctional cobalt phosphide/nitrogen-doped porous carbon composites. In situ generated reducing gases (CO, H_2_, PH_3_, etc.) from the nucleic acid were detected by hyphenated thermal analysis to evaluate EGA (TG-MS and TG-IR); also, they were suggested to be responsible for the transformation of phosphate in the precursor to phosphide in CoP. The exhausted sample could also be regenerated by a facile thermal treatment approach [74].

A series of Mn_0.15_Fe_0.05_/fly-ash catalysts have been synthesized by the co-precipitation method using coal fly ash as the catalyst carrier. The catalyst showed high catalytic activity for low-temperature selective catalytic reduction of NO with NH_3_. Various characterization methods were used to understand the role of the physicochemical structure of the synthesized catalysts on their De-NOx capability, like scanning electron microscopy, physical adsorption-desorption, and X-ray photoelectron spectroscopy. Furthermore, NH_3_/NO temperature-programmed desorption, NH_3_/NO-thermogravimetric-mass spectrometry, and in situ DRIFTs (Diffuse Reflectance Infrared Fourier Transform Spectroscopy) results showed that the Mn_0.15_Fe_0.05_/FA has relatively high adsorption capacity and activation capability of reactants at low temperatures [75].

Zhang et al. studied guard catalysts of the diesel hydrocracking unit in a refinery. They were collected after toluene extraction treatment and characterized by X-ray diffraction, carbon-sulfur analysis, EGA-mass spectrometry analysis, N_2_ adsorption-desorption, and SEM-EDX. The results showed that a large amount of Si, P, Fe, Ca, C and other impurities deposited and formed a “shell” over the guard catalyst, which blocked the pore channel and covered the outer surface of the catalyst, resulting in a significant reduction in the ability to contain impurities [76].

Evolved gases from AdBlue decomposition with different catalysts (Ti, Zr, Pt, SiO_2_, and γ-Al_2_O_3_) were analyzed with a thermogravimetric analyzer coupled to a mass spectrometer. The results showed that γ-Al_2_O_3_ is the best pyrolysis catalyst, and it promotes the decomposition of urea. The gaseous products were detected by MS at different reaction stages, resulting from the decomposition of urea and the formation of intermediate products [77].

Wu and coworkers synthesized the NiMoAl catalyst precursor by sol-gel method, and it was thermally treated in air, nitrogen and liquid paraffin, respectively, to prepare three kinds of NiMoAl catalysts. The results showed that larger pore volume and higher active metal dispersion were obtained in comparison with the sample calcinated in air [78]. Tetraammineplatinum(II) and tetraamminepalladium(II) chromates as precursors of metal oxide catalysts were reported by Filatov et al. [79].

Farooq et al. investigated the efficacy of a prepared Ni/θ-Al_2_O_3_ catalyst during the pyrolytic conversion of microalgae and compared the results with non-catalytic conversion. The presence of the catalyst facilitated the pyrolysis and the gases that evolved during pyrolysis were qualitatively analyzed to see the effect of the catalyst on evolved gas composition during the pyrolysis process [80].

A series of Ni-based CO_2_ methanation catalysts were prepared with different nickel salt precursors. The Ni-AA catalyst, with nickel acetylacetonate precursor, displayed the highest activity among these catalysts. EGA was employed to investigate the intermediates of the catalyst calcination process and allowed to establish that the significant role of the metal salt precursor should be preferentially considered when designing the Ni-based catalysts [81].

A circular economy becomes an object of actual discussions as a real alternative to the existing linear economy system. In the sector of heat and power production which is based mainly on the combustion of local solid fossil fuel, the thermooxidative decomposition of oil shale samples from different deposits and estimation of the possibilities of utilization of ashes formed at both pulverized firing and circulating fluidized bed combustion were studied by Kaljuvee et al. Evolved gas analysis methods like Fourier transform infrared and mass-spectroscopy was exploited and it was established that the differences in the behavior of different samples are caused by the differences in the chemical matrix of organic matter, the chemical and mineralogical composition, and the morphology of samples [82].

Co-pyrolysis of oil shale and organic solid wastes rich in hydrogen is significant for improving shale oil quality and recycling wastes effectively and cleanly. Transition metal salts were chosen to be catalysts for Jimsar oil shale pyrolysis by Pan et al. The pyrolysis behaviors of samples in the presence of different catalysts were compared using mass spectrometry to prove the nature of the evolved gases. The results and analyses allowed us to establish the optimal heating conditions for Jimsar oil shale pyrolysis [83]. The water content in shale is of great significance for shale gas and oil reserve estimations, and production predictions. Shen et al. proposed a facile and efficient method to quantitatively determine the status and relative content of water in shale [84]. Mu et al. applied a thermogravimetric system coupled with a mass spectrometry system (TG-MS) to investigate the pyrolysis characteristics of low-density polyethylene and polypropylene and explore their effects on the pyrolysis behaviors of oil shale during co-pyrolysis. The data from mass spectrometry indicated that the addition of plastics, especially the minor addition of plastic, increased the volatiles of medium molecular weight, which implied that gaseous reactions between volatiles from polyethylene and volatiles from oil shale [85].

EGA-MS helped in understanding the catalysts with low Ru loading in the combined CO_2_ capture and in situ methanation using renewable hydrogen over dual function sorbent-catalyst materials. The favorable synergism existing at the nanoscale between the Li-aluminate sorbent phase and the catalytic Ru sites enhances the intrinsic activity that can guarantee high methane productivity and selectivity with low noble metal loadings [86].

New magnetic Fe oxide-carbon-based acid catalysts were prepared by dissolution of iron salt in bio-oil followed by thermal treatment in a nitrogen atmosphere. Results from several characterization techniques showed that these materials can be used as catalysts for the esterification reaction of oleic acid with methanol with a good conversion factor [87].

Liu et al. studied the promoting effects that sulfate has on a NiMo/γ-Al_2_O_3_ catalyst and the promotion effect of Chromium on the activity and SO_2_ resistance of CeO_2_-TiO_2_ catalysts for the NH_3_-SCR reaction. Induced evolved gas analysis showed the hydrodesulfurization activity and proved that the catalyst has high metal dispersion and good stability [88,89]. Chen and coworkers used UV Raman, TG-MS (TPO), and nitrogen physisorption characterization to characterize deactivated Mo/HZSM-5 catalysts [90].

## 6. Applications to Flame Retardants

Following an innovative waste-to-wealth approach, humic acid was exploited as a flame retardant additive. The effect of its addition alone and in combination with urea and ammonium polyphosphate on the thermal, fire, and mechanical performances of a bisphenol A diglycidyl ether-based epoxy resin modified with (3-Aminopropyl)-Triethoxysilane and cured with aliphatic isophoronediamine has been investigated. The evolved gas, thermal, and fire analysis were used to propose the combined mode of action of humic acid, urea, ammonium polyphosphate, and silicon in the fire performance improvement of the hybrid epoxy system [91].

Chemical recycling is often used for the recycling of plastics included in waste electric and electronic equipment since fuels and secondary valuable materials can be produced. Brominated flame retardants are usually added to these plastics to reduce their flammability, but they are toxic substances. Charitopoulou and coworkers reported the thermal behavior and the products obtained after pyrolysis of polymer blends that consist of acrylonitrile-butadiene-styrene, high-impact polystyrene, polycarbonate, and polypropylene with a composition that simulates real waste electric and electronic equipment, in order to investigate the effects on pyrolysis products. Thermal degradation of the blends was described by mass spectrometric-based evolved gas analysis [92].

Starch, distarch phosphate, and hydroxypropyl distarch phosphate were used as “green” carbon sources with ammonium polyphosphate to prepare flame retardant reconstituted tobacco sheets by the paper-making process. The effect of these starch-based flame retardants on their thermal degradation and combustion properties was preliminarily investigated. Cigarette burning cone analysis also confirmed that the modified sheet had a lower temperature and smaller burning cone volume [93].

Novel flame retarded unsaturated polyester resins have been developed and prepared by the introduction of high nitrogen content additives into the polymer matrix in order to verify their effectiveness in the formation of swollen carbonaceous char inhibiting the burning process of the polymer. The volatile compounds that evolved during the burning of materials were determined using a steady-state tube furnace and a mass spectrometer. It was found that the incorporation of new intumescent flame retardants led to the formation of carbonaceous char layers inhibiting the decomposition process and limiting the smoke emission [94].

EGA-MS analysis of a fire-retardant based on the synergy of tris(1-methoxy-2,2,6,6-tetramethyl-4-piperidinyl)-phosphite and melamine hydrobromide/aluminum diethylphosphinate in polypropylene showed that the fire-retardant rating was improved from none to UL 94 V-2 and the total heat release and peak heat release rate dropped [95]. The unusual role of labile phenolics in imparting flame resistance to polyamide was reported by Yu et al. [96]

A facile methodology to durably modify cellulose fibers by forming in situ phosphine oxide macromolecular physical networks was developed by Nazir and coworkers. The in situ phosphine oxide network was integrated within the cellulose fibers to produce a durable treatment against laundering. Analysis of evolved gases during the thermal decomposition of treated cellulose supported both the condensed and gas phases mechanism. The flame retardant treatment was further adapted to include antimicrobial properties by in situ formation of silver nanoparticles within the cellulose fibers using a single-step process to demonstrate the versatility of this novel treatment methodology [97].

The transition from smoldering to flaming was studied by Morgan et al. on fabric, batting, and foam assemblies via an electric spot ignition source of similar intensity to a cigarette. The materials studied included different fabrics (cotton, polyester, cotton/polyester blend, flame retardant cotton/polyester blend), batting (cotton, polyester), and polyurethane foam (non-flame retardant and flame retardant by two different testings). The evolved gas analysis allowed us to compare the different behaviors [98].

Using depolymerized lignin as raw material, a new type of expanded lignin-based nitrogen and phosphorus flame retardant was proposed and characterized by Li et al. EGA-MS allowed the description of the characteristics of this innovative material [99].

## 7. Applications to Epoxy Resins

The effect of the addition of additives such as melamine, silica nanoparticles, and a phosphorus-based compound on the fire and mechanical performance of a bisphenol A diglycidyl ether-based epoxy resin cured with isophoronediamine was investigated by Bifulco et al. Evolved gas, thermal and fire analysis was used to propose the combined mode of action in the fire performance improvement of the epoxy system [100].

Castor oil, a triglyceride ester of ricinoleic acid containing up to 2.7 hydroxyl groups per molecule, was converted with three cyclic anhydrides into esters with carboxyl in their structures. The obtained products have potential as crosslinking agents for epoxy resins. The chemical structures, thermal properties, degradation mechanisms, and gaseous composition obtained during the thermal decomposition of the products in the nitrogen atmosphere were identified by the on-line coupled FT-IR and MS spectrometers [101].

## 8. Applications to Biomass

Biomass is an energy source that is abundant, clean, and renewable, and the development and utilization of biomass can effectively solve environmental pollution and energy problems. However, there are a series of problems such as the low calorific value of produced gas and oil that inhibited its exploration. Biomass pyrolysis has been the focus of study by several researchers as a viable means of producing biofuels and other useful products. Amenaghawon and coworkers gave a comprehensive, elaborate, and updated review of pyrolysis technology as an efficient thermochemical route for biomass conversion. Pyrolysis products included pyrolytic gas, bio-oil, and solid biochar. Depending on the operating conditions, pyrolysis is usually classified as slow, intermediate, fast, and flash pyrolysis. More advanced experimental methods have been developed for biomass pyrolysis research, and these include EGA-MS/FID, EGA-MS-FTIR, in situ spectroscopy for reaction progress analysis, isotopic labeling, and intermediate product analysis techniques that enable the monitoring of the biomass devolatilization process as well as identification of the functional groups of the volatiles and monitoring of the changes in the functional groups on the surface of biomass in the course of pyrolysis [102,103].

Tu et al. applied a two-stage method of hydrothermal carbonization and chemical activation technology to prepare a novel, large surface area and rich-pore structure activation-hydrochar from sludge sewage and coconut shell due to its mild, low-cost, and well-developed merits. The pore-making mechanism of activation-hydrochar was proved by several techniques, including TG-MS characterization. The results illustrated that the first stage of hydrothermal carbonization achieved the rich-pore structure hydrochar via dehydration, decarboxylation, deamination, and rearrangement reactions [104].

The unburnt carbon content of fly ash is an important index for evaluating the combustion efficiency of boilers. Measurement by traditional loss-on-ignition, approximate and elemental analyses, causes large errors due to mixing minerals such as portlandites, carbonates, or sulfates with the unburnt carbon. A new method of Equivalent Characteristic Spectrum Analysis (ECSA^®^) based on thermogravimetric-induced evolved gas analysis by Mass Spectrometry was adopted to determine the unburnt carbon in fly ash to provide reliable information to assess the operating status of boilers [105]. The effect of biogas plant waste on the physiological activity, growth, and yield of Jerusalem artichoke and the energetic usefulness of the biomass obtained in this way after the torrefaction process was reported by Szufa et al. [106]. The use of waste from corn grain biodigestion to methane as a biofertilizer caused an increase in the physiological activity, growth, and yield of Jerusalem artichoke plants and can limit the application of chemical fertilizers, whose production and use in agriculture is harmful to the environment. EGA-MS showed a thermo-chemical conversion mass loss at a level of 30% with energy loss (torgas) at a level of 10%.

Detailed analysis of thermodynamics, kinetics, and reaction products can provide valuable information about peanut shell pyrolysis reaction, which is essential for its efficient utilization. Tao and coworkers elucidated the pyrolytic reaction mechanism by the mass spectrometric analysis of the evolved gases and the structural characterization of the derived biochar. This study provided a prospective approach to understanding the pyrolysis mechanism of biomass [107].

Co-processing of biomass and heavy oil is believed a good way to reduce the difficulty of heavy oil processing and solve the problems of the low calorific value of the gas and oil produced from biomass. The pyrolysis and co-pyrolysis characteristics of corn stalk and a petroleum residue obtained from the fluid catalytic cracking process at different blending ratios were studied by Xiang et al. via thermogravimetry coupled with mass spectrometry. Combined with online mass spectrometric data, the amount of gas emission increases regularly with the increase of corn stalk addition, indicating that the change caused by the interaction of biomass and fluid catalytic cracking is mild during co-pyrolysis [108,109].

The utilization of biomass waste by biochar production seems to be an efficient and environmental friendly way of biomass treatment. Biochar is produced from biomass by low-temperature pyrolysis (torrefaction). Kwoczynski and Cmelik characterized twelve different biomass wastes generated in the Czech Republic by EGA-MS. Different from most published works, which focused on the analysis of evolved hydrocarbon gases or analysis of condensed liquids, they focused mainly on inorganic gases produced during pyrolysis, which can be toxic, explosive, or corrosive. Experiments suggested that the real conditions of pyrolysis on an industrial scale can be adjusted according to the EGA-MS data. These data can also be used for the selection of the most suitable biomass for pyrolysis, where almost no gases are generated to avoid corrosive effects on the pyrolyser [110].

Magida et al. investigated the combustion behavior and evolved greenhouse gases of coal, Scenedesmus microalgae, and coal-Scenedesmus microalgae blends (Coalgae^®^) in air using EGA-MS. Results confirmed that the combustion behavior of these materials was different. Mass spectrometric data presented a decrease in the emissions of CO_2_, NOx, and SO_2_ from coal to Coalgae^®^ due to the complex combustion mechanism in the presence of radicals [111].

The process of pyrolysis of beet pulp, a by-product after the extraction of raw sugar from sugar beet, with the addition of defecation lime was studied by Slezak et al. The resulting char, tar, and gas were characterized, and the addition of the defecation lime to beet pulp caused both an increase in the char production yield and a decrease in the tar production yield [112].

Gasification of the char obtained from the spent substrate after mushroom cultivation was carried out in a thermobalance connected to a mass spectrometer in the temperature range from 200 to 950 °C in an inert atmosphere. It was observed that the saturation of active sites on the char particles occurred above 50 vol% CO_2_, regardless of temperature, and the analysis of gas composition indicated CO as the main product of gasification [113].

During the pyrolytic production of biochar, only 50% of carbon could be retained in biochar, and about 10% would be mineralized after biochar is amended into soil. Nan et al. searched for a material to pretreat the biomass and improve carbon sequestration during biochar production and application, with the focus on alkaline minerals’ influence on biochar carbon sequestration. The presence of compounds from the thermal decomposition was verified by TG-MS to offer a potential strategy to strengthen biochar-carbon sequestration by metal-doping from the production of biochar to the application in the soil environment [114].

The biomass chemical looping gasification of torrefied spruce-pine-fir sawdust was studied using EGA-MS to investigate the effects of torrefaction temperature. The results showed the volatiles release intensity reached the maximum when the torrefaction temperature was 270 °C and that the torrefaction affected the distribution of the gaseous product [115]. Chemical looping combustion is an advanced technology for converting solid fuel with in situ CO_2_ capture in a low-cost and sustainable way. Zeng et al. focused on the solid reactions with gas intermediates taking place in the chemical looping combustion reactor. The effects of different particle size ranges and different reaction temperatures were determined and the result showed that the carbon gasification was identified as the control step in reactions [116]. Li et al. compared two methods to measure heterogeneous redox kinetics of fluidizing oxygen carrier particles by both the solid mass signal measured from the weight balance and the gas evolution signal measured by mass spectroscopy. Microfluidized bed thermogravimetry combined with the mass spectrometry method (MFB-TG-MS) was used to measure the tail gas signal, and heterogeneous redox kinetics was obtained from the analysis of the tail gas signal [117].

Ahmad and coworkers focused on the evaluation of the bioenergy potential of waste biomass of desert plant Calotropis procera. The pyrolysis reaction kinetics and thermodynamics parameters were assessed using isoconversional models, namely Kissenger-Akahira-Sunose, Flynn-Wall-Ozawa, and Starink. The evolved gases were dominated by propanoic acid, 3-hydroxy-, hydrazide, hydrazinecarboxamide, and carbohydrazide followed by amines/amides, alcohols, acids, aldehydes/ketones, and esters [118].

Radojević and coworkers provided in-depth knowledge about the evolved gas analysis interpretation via the newly proposed semi-quantitative approach, arising from thermogravimetric analysis coupled to mass spectrometry, for studying the pyrolysis behavior of three kinds of biomass waste materials (spent coffee grounds, beech sawdust, and wheat straw). TGA-MS coupling allows accurate correlation between molecular ion peak and fragment peaks to the corresponding mass-loss rates from derivative thermogravimetry curves. It was shown that, by this procedure which involves overlapping multiple curves supervising, the identification of gases in the volatiles complex scheme becomes more simplified. Easy and reliable calculations of gaseous product yield and syngas energy capacities are possible to achieve [119]. Babinszki et al. studied biomass wastes derived from palm oil production [120]. Catalytic conversion of pyrolysis vapor from biomass was performed over CeO_2_ catalysts to produce ketones, and TG-MS was applied to understand the distribution of pyrolysis vapors from various biomass including rape straw, poplar, cypress, and bagasse [121].

Thermochemical processes for biomass conversion are studied to produce renewable hydrogen-rich syngas. Model fitting methods were used by Quiroga et al. to propose thermal degradation kinetics during catalytic and non-catalytic pyrolysis (in nitrogen) and combustion (in synthetic air) of sugarcane residual biomass. Simultaneous FTIR-MS-EGA results showed three different degradation stages and a catalyst effect on product distribution [122].

The energy potential of buriti (*Mauritia flexuosa* L. f.) and inaja (*Attalea maripa* Aubl. Mart.) seeds for pyrolysis was evaluated by five isoconversional methods to determine the kinetic and thermodynamic parameters. In addition, the evolution of the gas during pyrolysis was detected to evaluate the possibility to convert it into energy and valuable chemicals [123,124].

Boric acid has been recently proved to be a good substitute for conventional acidic catalytic materials. Hou et al. used boric acid as a catalyst in biomass pyrolysis (woody biomass) and reported mass loss, composition, and distribution of evolved volatiles formed from the pyrolysis process. Boric acid significantly altered the composition and distribution of volatile pyrolysis products of wood flour. It significantly increased the contents of small molecule compounds such as acetic acid and furfural but, decreased the contents of phenol derivatives with high molecular weights and these changes became more pronounced as the temperature increased [125].

## 9. Applications to Oil and Bitumen

The chemical study of bitumen from stone tools from Italian Neolithic sites was carried out using analytical pyrolysis-based techniques to obtain evolved gas analysis by mass spectrometry. EGA-MS was employed to obtain information on the thermal complexity of the samples and on their thermal degradation areas, that is the set of thermal properties and behaviors. Geological bituminous rocks from Central-Southern Italy were selected and used as reference materials to both optimize experimental parameters and support data interpretation for archaeological samples [126].

A new Rock-Eval^®^ method (Shale Play™) has been proposed by Cadeau et al. It was widely used to identify the type and the thermal maturity of sedimentary organic matter, as well as for quantifying the total organic carbon content. Traditionally, it is a screening tool to estimate the petroleum generation potential of source rocks using standardized parameters. Cadeau’s group highlighted the investigation of the hydrocarbon content of liquid-rich tight rock samples. A dual vacuum and on-line system was proposed, in which thermally vaporized products were divided so that half was analyzed by the Rock-Eval^®^ FID while the other portion was cryogenically trapped in the on-line recovery system calibrated using known quantities of gas [127].

Oils, e.g., linseed oil, have been used as mortar admixtures or components of lime-oil mastic since ancient times. The reason was either to increase the mortar durability and/or to prolong the mortar/mastic workability. The goal of the research proposed by Bauerovà and coworkers was to evaluate the detection and possible quantification of linseed oil in a prepared mortar containing, beyond the oil, just lime, and calcite. With the help of Evolved Gas Analysis, the organic content analysis provided good information about the total amount of organics in mortar [128].

The effects of H_2_O and CO_2_ on the simultaneous removal of H_2_S and SO_2_ by activated carbon were studied. Fresh carbon samples and desulfurization-sulfur-loaded activated samples were characterized by several techniques including TG-MS [129].

The syngas distribution from steam gasification depends on both the feedstock and the gasification conditions. An easily applicable computational method for thermoanalytical behavior during steam gasification was proposed to predict the gasification behavior of steam gasification using the Artificial Neural Network as a machine learning approach [130]. Yu and coworkers investigated the effects of a series of additives ranging from alkali, alkali earth, transition, and rare earth metals on catalytic behaviors in steam reforming of acetic acid [131].

## 10. Applications to Cellulose, Lignin and Lignite

A 2021 review proposed by Lucejico et al. collected studies on the capability of analytical pyrolysis-based techniques to provide data on lignin composition and the chemical alteration undergone by lignin in archaeological wooden objects. Applications of Direct Exposure Mass Spectrometry (DE-MS), Evolved Gas Analysis Mass Spectrometry (EGA-MS), and single and double-shot Pyrolysis-Gas Chromatography/Mass Spectrometry (Py-GC/MS) in archaeological lignin characterization are described. It is underlined that, in comparison to cellulose and hemicelluloses, lignin is generally less prone to most degradation processes affecting archaeological artifacts in burial environments, especially waterlogged ones, which are the most favorable for wood preservation. Nevertheless, lignin also undergoes significant chemical changes. Analytical pyrolysis coupled with mass spectrometry, used in several complementary operational modes, gathered information regarding the chemical modifications and the state of preservation of lignin [132].

The potential of biomass pyrolysis to mitigate climate change needs to be reconsidered. Induced pyrolysis of beech, oak, and spruce wood was carried out to evaluate the course of pyrolysis, the conditions affecting the biochar yield, its fixed carbon content and yield, specific surface area, and gas-phase composition. The thermal degradation of the main wood components occurred at the same temperatures with a comparable gas composition determined by mass spectrometry evolved gas analysis. By the use of pyrolysis instead of combustion, CO_2_ emissions are reduced by almost half due to carbon sequestration through biochar formation [133].

Biomass-derived chemical looping gasification is a novel technology for lignocellulose energy applications. Ca–Fe oxygen carriers have been proven to be a potential material for efficient lignocellulose conversion and hydrogen-enriched syngas production in process studies. Tang et al. coupled thermogravimetry, pyrolysis, and fixed-bed to mass spectrometry to explore the mechanism of the reaction between Ca–Fe oxygen carriers and biomass char or volatiles. The mechanism of the synergistic effect of Ca–Fe was proposed on the basis of the EGA results [134].

Co-pyrolysis of biomass and plastics gives rise to synergistic effects that can improve the properties of the resulting bio-oil. Nardella et al. studied the co-pyrolysis of lignocellulose (softwoods and hardwoods) and plastic (polyethylene and polystyrene) mixtures by evolved gas analysis-mass spectrometry. By the shift of the peak temperatures and the differences between the integrated area of the two thermograms, it was shown that both biomass and plastic influence the pyrolytic behavior of the other component [135].

An electron impact ionization mass spectrometer was used to investigate the evolutions of gases and small molecular weight oxygenates during the pyrolysis of a raw lignite and a potassium-impregnated lignite by Yan and coworkers [136].

Transformations of aspen wood under the impact of ozone were investigated by simultaneous thermal analysis combined with mass spectrometry of non-condensable products of pyrolysis. Based on the obtained EGA/MS analysis, the range of ozone consumption corresponding to the predominant destruction of lignin and hemicellulose, and the range of ozone reactions with the ozonation products were established [137].

Mattonai et al. investigated the effects of ball-milling and UV irradiation on two species of softwood and two species of hardwood. The EGA-MS data and isoconversional kinetic analysis showed that both milling and irradiation can reduce the thermal stability of wood up to a limit value. Experimental results showed that UV irradiation can decrease the recalcitrance of biomass toward pyrolysis, but its efficiency is highly dependent on the type of lignocellulosic substrate [138,139].

The aggregate structural characteristics of Yimin lignite under heat treatment at 384, 456, 528, and 600 °C were examined mainly by HRTEM technique, and EGA-MS was additionally employed to measure the gaseous evolution characteristics from 30 °C to 900 °C. The results indicated that the length, crooked degree, orientation distribution, layer spacing, and stacking height of the aromatic fringes varied accordingly with increasing temperature and were closely related to the gaseous release characteristics during pyrolysis [140].

A series of metal oxides were employed by Li et al. to investigate the role of varied acid-base sites on kinetics and mechanism for catalytic fast pyrolysis of cellulose, waste wood, and soil humic substances. The results showed that the modulation of acidity-to-basicity value via altering metal oxides constituents significantly affected the transformation pathway for cellulose pyrolysis. Higher acidity-to-basicity ratios accelerated the proceeding of the deoxygenation process while lowering acidity-to-basicity ratios mainly facilitated the ketonization and aldol condensation [141,142,143].

By tracking the evolution of the carbonized products through mass spectrometry, in situ FTIR, and other characterization methods, the gas-exfoliation and in situ template functions of zinc carbonate and zinc oxalate were proposed by Fu and coworkers, who found that the pyrolysis synergistic effect boosts the decomposition of oxygen-containing groups, playing a pivotal role in the fabrication of hierarchical porous carbon with crumpled nanosheets [144].

## 11. Applications to Materials

Evolved gas analysis by mass spectrometry has been used to characterize the effect of heating a material in an oxidative or non-oxidative environment. Measurements in an oxidative environment are usually done by using the mixture of air and helium as the carrier gas; however, the ionization efficiency of evolved gases in a mass detector decreases in such an environment, resulting in a corresponding decrease in sensitivity. The drop in sensitivity can be reduced by diluting the air in the carrier gas with He after evolving gases from a sample and before the evolved gases flow to the mass spectrometer. Shiono et al. reported a simple modification of the gas inlet system so that experiments requiring oxidative conditions in an air atmosphere can be routinely performed without loss of sensitivity [145].

Temperature-programmed desorption and temperature-programmed reduction experiments were performed in a plug-flow reactor to analyze the decomposition mechanisms of oxygen-containing surface groups by monitoring evolved water, carbon dioxide, and carbon monoxide quantitatively [146].

The amount of adsorbed styrene acrylate copolymer particles on cementitious surfaces at the early stage of hydration was quantitatively determined using three different methodological approaches: the depletion method, the visible spectrophotometry, and the Evolved Gas Analysis by mass spectrometry (EGA–MS). The newly developed method, using EGA–MS for the quantification of particles, proved to be suitable for dealing with these issues. Here, instead of the fluid phase, the sediment is examined with regard to the polymer content, on which the influence of centrifugation is considerably lower [147].

Recent findings in nanomedicine have revealed that carbon nanotubes can be used as potential drug carriers, therapeutic agents, and diagnostics tools. Moreover, due to their ability to cross cellular membranes, their nanosize dimension, high surface area, and relatively good biocompatibility, carbon nanotubes have also been employed as a novel gene delivery vector system. Celluzzi et al. extensively investigated the chemical, biophysical and biological contributions of polyamine-coated carbon nanotubes [148]. Functionalized carbon nanotubes using two polyamine polymers, polyethyleneimine, and polyamidoamine dendrimer, were investigated by thermal analysis in order to address preparation strategies to obtain low cytotoxic compounds with the ability to conjugate microRNAs and, at the same time, to transfect efficiently endothelial cells [149].

The emission of volatile compounds from lignin-alumina hybrids, tested as effective additives for application in abrasive materials, mainly phenol and formaldehyde, was investigated using detailed evolved gas analysis performed by means of quadrupole mass spectroscopy (QMS). It was established that the addition of these hybrid additives can reduce the emission of phenol and formaldehyde [150].

SO_2_ emissions cause severe acid rain and smog. For coping with this terrible problem, melamine was proposed by Liu et al. as an absorbent for SO_2_ removal from simulated flue gas. By EGA-MS it was demonstrated that, compared with liquid organic amine, the solid melamine can avoid volatilization and reduce energy consumption during regeneration, indicating promise in industrial applications [151].

The study on the gas production behavior and pyrolysis kinetics of citric acid and tartaric acid provided a new idea for the researchers to choose a certain carboxylic acid. The results of EGA-FTIR-MS showed that, compared with tartaric acid, the activation energy required for citric acid pyrolysis is lower, and more reducing gas (CO) can be produced, which is more suitable for use as a reducing agent. Therefore, Wang et al. used citric acid among other raw materials to synthesize LiFePO_4_/C cathode materials by the carbothermal reduction method [152].

Bifunctionalized mesoporous silica materials were prepared by sol–gel method applying a newly proposed sequence of addition of the used silanols in the proposed systems. The bi-functionalized hybrid silicas were synthesized by co-condensation reaction and a soft template approach for pore formation was applied. The synthesized bi-functional hybrid mesoporous silicas were investigated by several techniques, including EGA-MS, and it was found that either the sequence of addition of silanol precursors, the type of the silsesquioxane precursors, and the presence of isocyanurate groups have a significant influence on the material’s texture, morphology and CO_2_ sorption properties, or the presence of isocyanurate groups in the hybrid silica framework significantly improves the textural characteristics [153].

The calcium silicate hydrate gel was synthesized by the double decomposition method because of the simplicity and the quickness of the procedure. Simultaneous thermodifferential–thermogravimetric analysis and mass spectrometry (DTA-TG-MS) was used to identify the amount of calcium carbonate formed due to the reaction between the calcium and atmospheric CO_2_. With DTA/TG/MS, mass loss was observed to take place at temperatures below 400 °C, unidentified to date, which might be associated with the CO_2_ adsorbed on the gel [154].

Lemougna and coworkers investigated the effect of organic resin contained in glass wool on the synthesis of alkali-activated binders. The study was performed on glass wool containing sugar or phenolic resin, comparing it with glass wool that did not contain resin, as a reference. The results showed that the organic resin could be qualitatively identified, with gradual decomposition occurring between 200 °C and 550 °C [155]. Using evolved gas analysis and combining the temperature-dependent melt viscosity with x-ray data, Luksic et al. described the evolution of foam structure during the foam growth and subsequent collapse [156]. Cement-free refractory binders, considered an alternative to calcium aluminate cements, were subjected to conversion from a dry mixture to a hydrated matrix to simulate the thermal behavior of the hydrated castable matrix. The thermal decomposition mechanism and microstructure were examined by coupled DSC-TG-EGA-MS [157].

Crosslinking is a common method for preparing biomass-based carbon materials. Corn starch grafted with epichlorohydrin was prepared by Li et al. and then stabilized and carbonized to obtain starch-based near-spherical carbon materials. Results indicated that for the modified starch the temperature at which the weight loss rate is at maximum is lowered so that the pyrolysis is relatively mild, the destruction of the basic skeleton is reduced and the carbon yield greatly increased compared with pristine starch [158].

The stabilization of isotropic pitch-derived fibers and mesophase pitch-derived fibers was performed by Peng and coworkers at different heating rates and with different final stabilization temperatures. The stabilized fibers and carbon fibers were characterized by elemental analysis, FT-IR, TG-MS, and SEM to investigate the influence of the degree of oxidation on the microstructures. Results showed that a slow heating rate during stabilization was beneficial to the oxidative cross-linking [159].

Kmita et al. compared the thermal destruction course of one of the commercially available binders applied in the ALPHASET technology independent of the temperature, heating rate, and the character of the atmosphere (inert, oxidizing, or reducing). The EGA system allowed for the analysis of gases evolved at very fast heating in the inert atmosphere (pyrolysis) as a function of temperature [160].

Kaolinite hybrid material, functionalized by the μ-oxo Fe^3+^-phenanthroline complex, was selected to test its ability to efficiently remove and store gaseous heptanethiol. Evolved gas analysis by mass spectrometry was employed to characterize the material before and after the exposure to the gas and to define the adsorption process [161].

Glow discharge-mass spectrometry and evolved gas analysis were employed by Gong et al. to analyze the main impurities in the millimeter-sized freestanding AlN single crystals with different colors. The results show that the content of oxygen in the light yellow crystals is lower than that of the dark brown crystals, while the carbon content is opposite in these crystals with different colors [162].

Two grades of high-speed steel, having different carbon content and processing treatment (i.e., cryo-milling and atomization), were used as a metallic binder for NbC cemented carbides. The results from evolved gas analysis during thermal heat treatment indicated that the processing treatment has a great impact on the thermal behavior of the steel powders, which later can be traced to the thermal behavior of the compacts [163]. The volatilization problem of mold fluxes significantly affects their physicochemical properties and then the quality of steel shells and the casting process. By studying the qualitative and quantitative analysis of volatiles through EGA–MS, XRD, and SEM–EDS, Gu and coworkers followed the mechanism of fluorine volatiles in mold fluxes during the casting process. The results showed that fluorine volatiles were formed when the temperature was higher than 1000 °C [164].

Highly porous cerium oxide in the form of easy-to-handle microspheres and particles of micrometric size was produced by the low-temperature calcination of Amberlite^®^ XAD7HP microspheres saturated with cerium nitrate solution. Among the other characterizations, the effect of temperature on the polymer microspheres saturated with cerium nitrate solution was assessed in synthetic air, in conditions resembling the calcination process in the furnace, by means of thermogravimetry with the simultaneous QMS analysis of the gases evolved during their degradation and also of DSC analysis [165].

The solid-state polymerization of organic molecules to form two-dimensional materials remains a challenge, especially when these reactions are performed in one pot using a single reagent. Relevant mechanistic aspects, such as the dehydrocyanation and deamination processes during solid-state polymerization of diaminomaleonitrile to obtain a new generation of conjugated functional materials were reported by Hortelano et al.This information allows us to propose hypothetical pathways for the production of complex conjugated systems, predominantly constituted by a 2D macrostructure [166].

## 12. Applications to Composites

Many researchers regard silica, silica-based, and zeolite membranes as the agents that will accomplish H_2_ separation. These membranes are expected to be productive in various mixture systems and under very high temperatures. Lawal and coworkers reported the successful fabrication of a composite carbon-SiO_2_-ZrO_2_ ceramic membrane with a unique pressure-induced switching of CO_2_ flows that allows versatile H_2_/CO_2_ separation at elevated temperatures. EGA-MS, FT-IR, CP-MAS-13C-NMR, and TEM provided corroborative evidence of the carbonization of starting material. This novel C-SiO_2_-ZrO_2_ material shows promise for many interesting applications [167].

A three-way EGA-MS-PCA was applied by Watanabe et al. to probe the interfacial state between matrix and filler in poly(styrene-b-butadiene-b-styrene) nanocomposites containing graphene nanoplatelets. The data sets are subjected to mathematical decomposition based on PCA to selectively derive essential information. The EGA-MS-PCA process reveals the formation of a π–π stacking interaction [168].

The dielectric and sorption properties of some flax fiber-reinforced ethylene-propylene-diene monomer composites containing different fiber loadings as well as their behavior after exposure to different doses of electron beam irradiation were studied by Airinei et al. Using thermogravimetry to induce evolved gas analysis by a simultaneous TG-FTIR-MS system, the degradation products were identified. Results mainly demonstrated that the water uptake increased as the flax fiber level increased in composites [169].

Organic-inorganic silica composites have been prepared by Putz et al. via acid-catalyzed sol-gel route. Thermal analysis has been used in order to evaluate both the thermal and structural stability of the materials (nitrogen and synthetic air atmosphere) and the decomposition mechanism inside the silica matrix (mass spectrometric evolved gas analysis). The measurements showed that the degradation of the longer side chain (butyl) starts at low temperature (above 150 °C) through a C-N bond cleavage, initiated by the nucleophilic attack of a fluorine ion [170].

The chitosan-cellulose composite fibers spun were characterized by a homogeneous distribution and a close packing of the two biopolymers inside the fibrous matrix. Due to the intimate contact of cellulose and chitosan, synergistic effects can be observed during the pyrolysis of the composite fibers. The catalytic role of chitosan in altering the cellulose pyrolysis pathway in these composite fibers was confirmed and the characterizations of the evolved gases during pyrolysis revealed that chitosan promoted cellulose dehydration and substantially decreased the formation of levoglucosan, explaining the higher char yield [171].

High-entropy ceramic matrix composites were reported for the first time. Based on the systematic study of the pyrolysis and solid-solution mechanisms of the precursor by Fourier transform infrared spectroscopy, TG-MS, and XRD, the composites with uniform phase and element distribution were successfully fabricated by precursor infiltration and pyrolysis [172,173].

In the field of non-oxide ceramic composites, and by using the polymer-derived ceramic route, understanding the relationship between the thermal behavior of the preceramic polymers and their structure, leading to the mechanisms involved, is crucial. To investigate the role of Zr on the fabrication of ZrC–SiC composites, linear or hyperbranched polycarbosilanes, and polyzircono-carbosilanes were synthesized. The materials were characterized by several techniques, including EGA-MS, X-ray diffraction, and scanning electron microscopy [174].

A W modified MnFe/Ti composite was proposed and tested for low-temperature SCR of NOx EGA-MS results and DRIFTS spectra revealed that the oxidation of SO_2_ can be inhibited by restraining the transfer of electrons, which reduced the formation of metal sulfates [175].

Polymer-derived SiOC-C composites are typically obtained through pyrolysis of a polysiloxane precursor in an inert atmosphere. A study from Campostrini and coworkers showed that novel SiOC microstructures and compositions can be obtained when the pyrolysis is carried out in a reactive environment [176].

Energetic composite fibers were prepared by Zhai et al. using an electrospinning technique. EGA-MS results showed that the decomposition process of the composite fiber is fast, and the main decomposition products are nitrogen, CO, NO, and water. Additionally, if compared to the raw material, the composite fibers have a lower impact and friction sensitivity [177].

## 13. Applications to Nanoparticles and Nanomaterials

Komarkova et al. described the thermal behavior of nanotitania precursors and the influence of various amines and peroxide treatments on the properties of TiO_2_. The thermal degradation of amine-containing amorphous (peroxo)titanates was examined by evolved gas analysis by mass spectrometry in an inert and oxidizing atmosphere. In argon, the samples underwent a two-step degradation process, involving the release of moisture and decomposition or evaporation of amine, while in air conditions the organic component could be oxidized in an additional third step at even higher temperatures. EGA confirmed the presence of the original amine in the amino-titanates, while the organic parts that reacted with oxygen evolved from the peroxide group to form oxidation products [178].

Innovative preparation strategies for nanomaterial functionalization were proposed to provide a novel tool to be used as drug delivery vectors for biomedical applications [179]. In particular, three different carbon nanotubes were considered (very small, carboxylated, and buckypapers) and two polymers were used to study the functionalization. Different preparation procedures were investigated, including the selection of the most performing polymer to be linked to the nanomaterial. To simultaneously evaluate all the variables, an experimental design was planned and the recorded data were processed coupled with chemometrics to identify the preparation procedure providing new nanomaterials able to conjugate microRNAs and efficiently transfect endothelial cells [180,181]. Employing EGA-MS and TEM characterizations, Liang et al. found that carbon nanotubes which could keep catalyst activity were more prone to form on large nickel particles, while encapsulated carbon species that led to deactivation were inclined to deposit on small particles [182].

New nanocomposites were obtained via casting/evaporation method, and the evolved gas analysis of the products of the nanocomposite resulted in lower than those of pure polyvinyl alcohol. For the first time, flame retardancy was investigated using a microscale combustion calorimeter [183].

In order to develop a usable low-cost nanocomposite with better catalytic performances for solid propellants, the CuFe_2_O_4_/g-C_3_N_4_ nanocomposite was designed and successfully prepared by in situ solvothermal method. The thermal decomposition stages and related products were probed by mass spectrometry evolved gas analysis and revealed that nano g-C_3_N_4_-based metal composite oxides can be used as combustion catalysts and could be a more viable strategy in the field of solid propellants [184]. The thermal behavior, morphology, and antibacterial properties of silica/quercetin nanocomposite materials prepared by the sol–gel route were studied by Catauro et al. [185]

A facile approach was developed by Wang and coworkers to directly synthesize carbon-supported metal nanoparticles with pomelo peel as the carbon source and metal nitrate solution as the metal source. Fe/C, Co/C, Ni/C, and Cu/C catalysts were prepared after calcination in nitrogen without further reduction. The metal nanoparticles and formation mechanism were investigated by multiple techniques such as XRD, HRTEM, XPS, FTIR spectra, SEM, and TG-MS thermal analysis. In the case of furfural hydrogenation, the Cu/C catalyst exhibited the best activity and exclusive selectivity to produce furfuryl alcohol with complete conversion, and excellent selectivity can be maintained at a higher temperature of 240 °C [186].

NiFe-carbon magnetic nanocomposites prepared using hybrid sebacate intercalated layered double hydroxides as precursors were shown to be of interest as supercapacitors. The low-temperature formation mechanism of these materials has been deciphered by means of a combined study using complementary in situ (temperature-dependent) techniques including EGA-MS. The experimental results confirmed the early formation of iron-nickel nanoparticles, preceding the concerted collapse of the starting laminar structure, leading to the formation of the final carbon nanocomposite [187].

A novel nanocomposite of Lanthanum-oxide-cluster-modified Ni nanoparticles loaded on mesoporous silica was synthesized and characterized by Zhang et al. The results of EGA-MS, TEM, XRD, isotope labeling experiment and Raman showed that oxygen causes a substantial reduction in carbon deposition rate, thus promoting the catalytic activity and durability [188].

The preparation of ordered mesoporous carbons containing uniform dispersions of ruthenium nanoparticles by a hard-template method was reported by Rio and coworkers. The catalysts were carefully characterized. EGA-MS contributed to comparing the catalytic activity in the liquid-phase hydrogenation of various substrates and their reusability and robustness [189].

## 14. Applications to Zeolites

A thermogravimetric analyzer connected to a mass spectrometer (TG-MS) characterized the in situ mass change that a zeolite particle underwent during the thermoanalytical run, while the MS monitored the decrease in C_2_H_2_ concentration and the appearance of C_2_H_2_ decomposition products (H_2_, CH_4_, C_6_H_6_) [190].

The catalytic behaviors of Ca-modified zeolites during the oil shale pyrolysis process were investigated in a tubular reactor and by EGA-MS-FTIR. The results showed that the molecular sieve can significantly increase yields of C1−4 aliphatic hydrocarbons and reduce their evolution temperatures. After modification, Ca-zeolites can reduce yields of CO_2_, increase yields of shale oil and decrease lengths of aliphatic chains in shale oil. However, Ca-zeolite has a strong catalytic effect on aromatization. Brönsted acid sites have an obvious catalytic effect on aliphatic hydrocarbons, while Lewis acid sites are more targeted at the aromatization process of pyrolysis products [191].

The liquid-solid post-synthetic method is commonly used in the preparation of metal-containing zeolites, for example, Ti-β (BEA) zeolite. Liang et al. reported a facile and Ti incorporation improved liquid-solid impregnation method for Ti-β zeolite preparation by incorporating a unique Ti precursor. Among the other characterization techniques, EGA-MS allowed defining of the function mechanism of this unique Ti precursor [192].

## 15. Applications to Clays

Mineralogical characterization of clays used in the manufacturing of traditional ceramic products is critical for guaranteeing the quality of the final product, but also for assessing the environmental impact of the industrial process in terms of atmospheric emissions since the presence of impurities even in low-level concentrations can have a big impact. To carry out an accurate mineral quantification of those materials, which are related to carbon dioxide and acid emissions (hydrogen fluoride, hydrogen chloride, or sulfur dioxide), Silvero and coworkers developed the application of hyphenated techniques with mass spectrometry and Fouriertransform infrared spectroscopy to provide more valuable information and lower limit quantification than other primary techniques, such as X-ray diffraction or infrared spectroscopy. They developed a specific analytical procedure using evolved gas analysis to identify and quantify minerals such as chlorides, sulfides, carbonaceous materials, and minor clay minerals, including the analysis of acid emissions during the ceramic firing treatment [193].

Bentonite buffers at temperatures beyond 100 °C could reduce the amount of high-level radioactive waste in a deep geological repository. However, it is necessary to demonstrate that the buffer surrounding the canisters withstands such elevated temperatures while maintaining its safety functions (regarding long-term performance). For this reason, Kaspar et al. arranged an experiment with thermal loading of bentonite powder at 150 °C. The paper reports the changes that the Czech Mg/Ca bentonite underwent during heating for one year. These changes were examined by X-ray diffraction, evolved gas analysis, aqueous leachates, Cs sorption, and other techniques. It was concluded that montmorillonite was partially altered [194].

## 16. Applications to Coal

Coals are composed of macerals, mainly vitrinite (V) and inertinite (I). V and I were reported interacting with each other in pyrolysis in some studies but not interacting with each other in other studies. Cheng and coworkers studied the interaction of V and I in pyrolysis by induced evolved gas analysis coupled online with a mass spectrometer using blends of V and I prepared from 3 coals. The extent of V and I interaction is discussed based on the differences in characteristic parameters of TG-MS data and the radical concentration of chars determined by electron spin resonance. The V and I interaction occurs mainly in the temperature range of 400−600 °C where the majority of devolatilization takes place [195,196]. Xian et al. investigated the release behaviors and reaction reactivities of gaseous products during CO_2_ gasification of three chars with different sulfur contents [197].

The modeling of pyrolysis can help to understand, to predict, and optimize many industrial processes. Ma and coworkers proposed an improved parallel reaction model which tackles the issues of precision and rationality of the popular DAEM (distributed activation energy model). The model was established by optimizing the parameters of sub-reactions, which were estimated via analyzing the evolved gases by both MS and FTIR on-line coupled, to obtain experimental data of bituminous coal. The proposed parallel reaction model provided more accurate predictions of coal pyrolysis [198].

The application of three kinds of on-line pyrolysis coupled with mass spectrometry in coal pyrolysis was reviewed and advantages and disadvantages were compared. The evolution behaviors of primary products and the catalytic pyrolysis can be also implemented [199,200]. Studies on non-isothermal kinetics and the influence of calcium oxide on hydrogen production during bituminous coal pyrolysis were proposed by Zhang et al. [201] Low-temperature pyrolysis behavior of four low-rank coals from the biggest deposits of Mongolia was studied by Alyeksandr et al. using a fixed-bed reactor and Gray-King retort [202].

The chlorine content and the release behavior of chlorine in the air atmosphere of Wanxiang coal from Hami, Xinjiang, were studied by Ning et al. The distribution characteristics of chlorine in raw coal and its combustion products were explored by means of water washing, density classification, EGA-MS, XPS, XRD, and SEM-EDS. The volatilization and migration of chlorine in raw coal during combustion were preliminarily revealed [203,204]. Zhu et al. investigated the effects of the minerals on the structures and pyrolysis behaviors of three middle/low-rank Xinjiang coals that were mildly pretreated with a weak organic acid at a low concentration [205].

Co-pyrolysis characteristics of Zhundong coal and corn stalk mixtures were studied by means of EGA-MS and in situ DRIFT to analyze the release of the main small-molecule gas products and to study the effects of functional groups on the release of gas products. Results show that the co-pyrolysis components of Zhundong coal and corn stalk interact with each other [206].

Coal gasification using chemical looping has been suggested as an innovative coal gasification technology for producing high-quality synthesis gas with lattice oxygen from oxygen carriers instead of molecular oxygen. EGA-MS analysis indicated that direct solid-solid contact reaction between coal and oxygen carriers could not be negligible in the process [207]. The release of nitrogen from a chemical looping gasification system was investigated by Li et al. and the migration mechanism of gas-phase nitrogen was online characterized by evolved gas analysis [208].

Five coal samples from different origins were characterized for their pyrolysis and combustion features using coupled TG-MS. The main volatile and combustion products release were estimated using online 3D measurements of MS, based on their relative intensities and relevancy [209].

Mixing metallurgical sludge with pulverized coal for blast furnace injection can realize the utilization of sludge resources and improve the combustion efficiency of pulverized coal. The combustion characteristics and gas release characteristics of blast furnace pulverized coal added with sludge were studied by EGA-MS to explore the influence of the organic and inorganic components of sludge on the combustion process of blast furnace injection of pulverized coal. The results showed that the composition of sludge is mainly organic substances such as lubricating oil and inorganic substances; adding sludge can reduce the ignition temperature of pulverized coal and the temperature corresponding to the maximum combustion rate to improve the reactivity [210].

The apparent activation energy of coal samples during pyrolysis in situ loaded on was calculated. The results showed that metal salts promote the devolatilization process with the activation energy decreased. The catalytic effect on the evolution of hydrogen and methane was also studied via EGA-MS tests [211].

To study BTX production for bituminous coal pyrolysis, Shi et al. used a tube furnace reactor and an EGA-MS system to study the pyrolysis characteristics of Ningxia bituminous coal with and without a catalyst [212].

The analysis of pyrolytic decomposition of different metallurgical coals used in steel industries was carried out by Kundu and coworkers for a better understanding of the coking mechanism during the carbonization of coking coals. Experimental results showed that the addition of resin to coal enhanced fluidity and thermal cracking accompanied by an aromatization and poly-condensation process. It reveals the mechanism and characteristics of gases evolved and, in some cases, also helped to eliminate the discrepancy occurring for the same molecular weight components [213].

Nitrogen oxide (NO) is one of the major pollutants generated in the process of coal thermal conversion and shall be controlled in order to fulfill the requirements of industrial production and environmental protection. The ratio of oxygen concentration to carbon dioxide concentration (O_2_/CO_2_ ratio) is one of the key factors that affect the thermal coal-N transformation and nitrogen oxide generation. Ding and coworkers adopted the coal self-preheating combustion technology to obtain the high-temperature coal char on a bench-sale test rig, and combustion and gasification experiments of this modified fuel were carried out on a TG-MS combined analyzer [214].

MS and FTIR evolved gas analyses revealed that SO_2_ was the main gas produced, and the maximum SO_2_ gas evolution temperatures of coal samples were similar to the thermoanalytical peak temperatures. The gas products in the samples varied mainly in quantity but not in species [215].

The co-pyrolysis characteristics of cow manure and Meihuajing bituminous coal blends were investigated in detail. The mass loss behavior and gas evolution characteristics of the blends were analyzed by online mass spectrometry evolved gas analysis, and kinetic analysis was also performed [216,217].

Potassium carbonate was employed as an effective catalyst to explore the catalytic pyrolysis characteristics of Shenfu bituminous coal. The online investigation of the effects of catalyst loading amounts and pyrolysis temperature on the gas release characteristics was conducted using Fourier transform infrared spectroscopy and mass spectrometry evolved gas analysis. The results showed that potassium carbonate could increase the yields of pyrolysis gaseous products and reduce the peak temperature for gaseous product release [218].

A comprehensive analysis of HSW vitrinite coal pyrolysis was proposed by Bai et al. The results showed that the weight loss rate of pyrolysis decreased with an increased heating rate [219]. The behavior of vitrinite and inertinite of Qinghua coal at different pyrolysis temperatures and the changes in key gas components were investigated by Mao and coworkers. Furthermore, the differences in pyrolysis behavior between vitrinite and inertinite were discussed from the point of view of chemical bonding breaking and pyrolysis kinetics based on the Coats-Redfern model [220].

In order to inhibit the active free radicals in the oxidation process of coal at low temperatures, the use of catechin as inhibitors was proposed. EGA by simultaneous FTIR-GCMS system has been useful in determining the nature of the gaseous products that evolved during the heating of raw coal and coal-catechin samples. Compared with raw coal, the content of carbon oxide gaseous products released from the coal-catechin sample was significantly reduced at the same temperature [221].

## 17. Applications to Membranes

The separation of azeotropic mixtures such as methyl acetate and methanol via a membrane is an interesting and challenging issue since the membrane must be able to withstand these harsh solvents and provide good flux and selectivity. SiC-based membranes have attracted a great deal of interest due to their high mechanical strength, structural stability, and corrosion resistance at elevated temperatures. Wang et al. described the first use of polytitanocarbosilane, which is known as a precursor of continuous Si–Ti–C–O based membranes for the pervaporation removal of water or methanol. The physical and chemical properties during pyrolysis were characterized via EGA-MS, ATR-FTIR, XPS, XRD, and N_2_ adsorption-desorption isotherms [222].

## 18. Applications to Coatings

An innovative coating material to extend the shelf-life of fresh-cut pineapple classified as “minimally processed foods”, was proposed by Gullifa and Materazzi. The novelty of this work consists of the use of biodegradable cases for the storage of fruits during experiments under refrigerated conditions. In addition, the application of the coating process was evaluated over a period of 15 days and complete characterization of the evolved Volatile Organic Compounds (VOCs) was performed by EGA-MS to assess the effect of the coating material on the flavor, the appearance, and the quality of the fruits. Results demonstrated that the application of carboxymethyl cellulose and ascorbic acid on pretreated fresh-cut pineapple is able to reduce the aging process and prolong the shelf-life of pineapple without requiring conventional PVC cases for storage [223].

Chemical modifications in the chitosan structure may result in obtaining a new material with improved chemical properties, such as an ability to encapsulate lipophilic compounds. De Carvalho and coworkers synthesized cinnamic acid grafted chitosan nanogel to encapsulate the essential oils of *Syzygium aromaticum* and *Cinnamomum* ssp., in order to develop a material to be applied in the control of dermatophytosis caused by the fungus *Microsporum canis*. The EGA-MS technique helped to determine the specific characteristics of this new material with potential application in the control of dermatophytosis caused by the fungus *M. canis* [224].

## 19. Applications to Archaeology and Heritage

Faience objects produced from the fourth millennium BC in ancient Egypt are considered the first high-tech ceramics in human history. Despite extensive studies on manufacturing technology, many aspects of this complex technology remain a mystery and there is no methodology in place to unravel the techniques of Egyptian faience object production. Many techniques, including EGA by coupled STA-MS, focused on the object of this innovative investigation, a hemispherical faience bowl discovered by archaeologists excavating a Ptolemaic workshop district at the site of Tell Atrib in the southern Nile Delta. The multiproxy analysis included the application of specialized software and preparation techniques coupled with complementary methods of light and digital microscopy. The results helped to reconstruct how the raw material was prepared and how faience vessels were made [225].

The first synthetic polymers were introduced as constituents of everyday life, design objects, and artworks at the end of the 19th century. Since then, the history of design has been strictly connected with the 20th-century evolution of plastic materials. Objects of design from the 20th century are today a precious part of the cultural heritage. They raise specific conservation issues due to the degradation processes affecting synthetic polymer-based plastics. Museums and collections dealing with the conservation of design objects and modern materials need to base their conservation strategies on compositional data that reveal the formulations of historical plastics and their decay processes. Specific and specifically optimized analytical tools, like evolved gas analysis coupled with mass spectrometry (EGA-MS) to characterize “historic polymeric materials” and heritage plastics at the molecular level with high chemical detail, were proposed to obtain significant or complete information on the nature and the state of preservation of the materials under study [226]. On the other hand, celluloid in museum collections is very unstable; therefore, heritage professionals carry out research studies dedicated to understanding its decay and prolonging its lifetime. Elsasser and coworkers addressed the need to compare and select suitable analytical methods for that purpose. Evolved gas analysis–mass spectrometry, double shot–gas chromatography/mass spectrometry, and gel permeation chromatography (GPC) were employed to characterize the emission of gasses (decay products) and measure the molecular weight and camphor (plasticizer) content from unaged, artificially, and naturally aged celluloid samples [227].

With the aim of consolidating heritage bricks, Navarro-Moreno et al. studied the strengths and drawbacks of nanolime, ethyl silicate, and sodium silicate coatings. The synergy of X-ray diffraction, X-ray fluorescence, and EGA-MS showed that the bricks are particularly rich in carbonates, although they also contain quartz and minor proportions of halite and gypsum, and that the consolidants based on sodium silicate formed hard coatings that were prone to cracks and efflorescence formation after a few days of curing [228].

## 20. Applications to Environment and Health

Environmental pollution from microplastics and the associated health and environmental risks are the focus of intense multidisciplinary research. There is an urgent need to produce quantitative and qualitative data on the amount and types of microplastics in environmental matrices, organisms, and commodities, and to perform spatial and temporal comparisons. This has led to the development, optimization, and application of analytical methods to characterize microplastics in environmental or biological samples. Instrumental analytical techniques based on analytical pyrolysis and thermal analyses provide qualitative and mass-based quantitative information and have a high potential to become of general use in the analysis of microplastics. La Nasa et al. reviewed the research carried out to date in the analysis of microplastics by analytical thermal and pyrolysis techniques [229,230,231].

Lee et al. demonstrated the relationships between elemental carbon and elemental carbon fractions during evolved gas analysis for PM_2.5_ sampled from March to May 2018. The elemental carbon concentrations were compared to the concentrations of equivalent black carbon to confirm that the concentrations analyzed in this study were valid. Dominant carbon fraction was related to the dependence of carbon oxidation quantity on the oxidation temperature. Authors reported, as absolute innovation, the correlation between pyrolytic carbon and the split time under NIOSH 5040 protocol with regard to EGA, helping to identify what causes uncertainty in the quantification of pyrolytic carbon [232].

The conversion of solid waste into energy through combustion must be environmentally sustainable. A study by Guo et al. aimed to comprehensively evaluate and quantify the co-combustion characteristics, synergistic catalysis, and gaseous pollutant emission patterns of sewage sludge and coal gasification fine slag residual carbon as well as their blends. The EGA-MS results showed that the co-combustion can not only improve the ignition and burnout property but also maintain the combustion stability and comprehensive combustion performance at a better level [233].

Environmentally dangerous microplastic particles are currently detected in almost all compartments. Detection methods vary widely due to very different requirements for analytical validity. Goedecke et al. compared and discussed the advantages and limitations of microplastic detection by EGA-MS techniques that are less common methods in this field [234].

The predictive potential of thermogravimetry in conjunction with multivariate statistical analysis was investigated for the screening of Sickle Cell Anemia, a hereditary disorder of hemoglobin characterized by severe hemolytic anemia with different severe clinical manifestations and hereditary erythrocyte membrane defects [235,236,237,238,239,240,241,242,243,244,245,246].

Due to a significant increase in the number of patients receiving chemotherapy, the development of reliable and fast analytical methods to assess the occupational exposure of workers in the manufacture of pharmaceutical products is fundamental and was applied for the first time as a novel approach for occupational exposure monitoring in the pharmaceutical field [247].

An innovative approach in thanatochemistry in order to develop a new analytical tool using thermal analysis for the characterization of vitreous humor was proposed. Vitreous samples were selected from medicolegal deaths which occurred in casualty and where the death interval is known [248,249].

Microwave pyrolysis for integrated with in situ reforming was proposed for clean syngas production from herb residues, a typical industrial biomass waste that has high moisture and nitrogen content. Effects of microwave heating, reaction temperature, and types of catalysts were investigated, and the tar elimination and nitrous oxides controlling performance were evaluated [250].

The combustion characteristics of alcohol extracted herb residue, wasted activated cokes and their mixtures with different proportions at different heating rates were studied by Zhu et al. The results showed that the combustion of alcohol extracted herb residues was a continuous process of volatiles and a small amount of fixed carbon, while that of wasted activated cokes was mainly a process of fixed carbon combustion. The EGA-MS system was used to compare the flue gases produced from the combustions of alcohol extracted herb residues and their mixture. The results showed that the addition of waste-activated coke has a significant adsorption and reduction effect on the NOx produced during the combustion of alcohol-extracted herb residue [251].

Risk control of Tibet herb residue through environment-sustainable disposal is in urgent need since Tibet is environment-ecologically vulnerable with very weak self-adjustment ability. Gasification is an effective way to dispose of Tibet herb residue with product gas, which could meet the energy demand of local users. EGA-MS and a tube furnace indicated most syngas were produced at 250–380 °C, and the reactions were slowed down under the Tibetan atmosphere. Results provided insights into using gasification for treating herb residue and even the environment-sustainably of other wastes in Tibet [252,253].

The recovery of phosphate as struvite from dairy lagoon wastewater limits contamination of surface water. Magnesium addition is used to promote struvite precipitation and suppress the formation of undesired phosphate mineral products. Simultaneous thermal analysis with evolved gas analysis (STA-EGA) showed high Mg concentrations decrease the sorption of micronutrients such as zinc and iron with recovered solids and indicated a potential benefit to the application of lower Mg concentrations [254].

Porous carbons prepared from biomass by the traditional method of caustic potash etching are common VOCs adsorbents, but their lack of mesopores hinders the mass transfer, limiting their application. Cheng and coworkers prepared templateless micro-mesoporous carbon with a high specific surface area by coupling Phanerochaete chrysosporium pretreatment and caustic potash etching and explored its synergy mechanism. The prepared micro-mesoporous carbon has simultaneously high specific surface areas and proportions of mesopores. The promoting effect determined by EGA-MS implied that P. chrysosporium treatment increases surface oxygen-containing groups [255]. Nitrogen and/or sulfur-doped carbons were extensively investigated as CO_2_ adsorbents. In a work published by Cao et al., potassium was used as a dopant for carbon and a new series of materials, namely potassium intercalated carbons, were prepared sequentially by the oxidation of mesoporous carbon, potassium ion exchange, and calcination. Based on results from FT-IR, XPS, and TG-MS, it was found that the pre-loaded potassium interacted with the carbon matrix during calcination, and the adsorbent showed fast and stable CO_2_ uptake in flue gas conditions, among the highest for carbon-based adsorbents [256].

In circular concrete design, besides cement replacement with more environmentally friendly cement types, there is also an urgent need for sand replacement with fine recycled concrete aggregates. An in-depth characterization of different Dutch aggregates was performed in order to examine their suitability as an alternative material for river sand and to define quality indicators. Their chemical analyses included quantification of element composition and carbonate content with EGA-MS [257].

## 21. Applications to Sewage Sludge

Pyrolysis and combustion have become effective technologies for oily sludge treatment. Chen et al. investigated N/S/Cl pollutants during pyrolysis and combustion of oily sludge in detail to propose releasing routes. Three typical decomposition steps were observed [258].

The co-pyrolysis technology of the second-generation feedstocks has both engineering and environmental advantages for resource recovery, waste stream reduction, and energy generation. However, there exists a large knowledge gap about the co-pyrolytic mechanisms, kinetics, emissions, and products of biomass wastes. This study aimed to quantify the co-pyrolytic interactions between different atmospheres and the two feedstocks of sewage sludge and coffee grounds as well as their emissions and products. EGA-MS analyses yielded valuable insights into a cleaner co-pyrolysis process [259].

Sewage sludge dehydration using skeleton conditioners is the main method of sludge treatment; however, the effects and mechanism of skeleton conditioning on pyrolysis products in the sludge conditioning–dehydration–pyrolysis process are still unclear. Pan et al. applied TG-MS and pyrolysis coupled to gas chromatography/mass spectrometry (Py-GC/MS) to characterize the release characteristics of condensable and non-condensable gases in the pyrolysis process of sludge samples treated with biochar, rice husk, and coal ash. The results indicated that the three skeleton conditioners did not noticeably promote or inhibit the release of S-containing pollutants during sludge pyrolysis [260].

To reduce the energy consumption during pyrolysis, the sludge is subjected to pretreatments like conditioning and dewatering, to reduce the water content. EGA-MS and pyrolysis–gas chromatography/mass spectrometry were applied by Wang et al. to clarify the release characteristics of condensable and non-condensable gases during pyrolysis of the sludge samples [261].

To elucidate the effects of iron oxide on nitrogen transformation during sludge pyrolysis, the influences of Fe_2_O_3_ on the pyrolysis characteristics, and the release of important gaseous NOx precursors such as HCN and NH_3_ during pyrolysis were studied. The results showed that the total weight loss rate and the initial decomposition temperature are reduced. The generation of Fe-N complexes reduces the generation of NH_3_, HCN, CH_3_CN, and HNCO [262].

Wang et al. introduced wood sawdust to improve combustion performance and inhibit the sulfur emission during dyeing sludge incineration. The combustion characteristics and release behavior of sulfur gas products of several mixtures were investigated. Results indicated that wood sawdust addition restrained the devolatilization of light volatiles and facilitated the degradation of heavy volatiles and fixed carbon [263].

## 22. Applications to Sulfur

Sulfur-containing pollutants are released during coal thermal conversion processes and must be controlled to satisfy the requirements of industrial production and protect the environment. The release behaviors of sulfur species were studied and reported by several authors.

Zhundong coal during combustion and gasification, and Shenmu coal were investigated as reference samples to obtain general release properties. The experiments were carried out using EGA-MS analysis and proposed theoretical calculation of the released gas. The results showed that the water washing process did not change the combustion reactivity and the sulfur release temperature. Controlling the temperature is helpful in reducing the release of SO_2_ in both combustion and gasification [264].

Zhao et al. reported an effective strategy to process high-sulfur residue oil while reducing the coke yield and simultaneously controlling the sulfur content of coke. The evolution of sulfur species in the integration process was investigated by XPS and online coupled TG-MS analysis. The process optimized the conversion of the refractory thiophenic sulfurs into sulfidic sulfurs as indicated by the increasing abundance of sulfidic sulfur, which thereby helped to reduce the sulfur content in coke. Moreover, it was demonstrated that the saturation of condensed-aromatic-ring structures of some refractory thiophenic sulfurs is promoted and thus the thermal cracking of these sulfurs [265].

Coal combustion releases elevated amounts of pollutants into the atmosphere, including SO_X_. During the pyrolysis step, sulfur present in the coal is released to the gas phase as many different chemical species such as H_2_S, COS, SO_2_, CS_2_, thiols, and larger tars, also called SO_X_ precursors, as they form SO_X_ during combustion. Understanding the sulfur release process is crucial to the development of reliable kinetic models, which support the design of improved reactors for cleaner coal conversion processes. The sulfur release was studied by Debiagi et al. either by low heating rate experiments, with EGA-MS allowing to track the mass loss and the evolution of many volatile species, or by high heating rate experiments in an entrained flow reactor (drop-tube reactor—DTR) coupled with MS and a nondispersive infrared sensor. This combination revealed the complexity of sulfur chemistry in coal combustion and allowed us to better understand the individual phenomena resulting in the formation of the different SO_X_ precursors [266,267]. Yu et al. determined the temperature-resolved evolution and speciation of sulfur during pyrolysis of a high-sulfur petroleum coke [268].

A series of monometallic and bimetallic Cu/Mg oxide-based structured catalytic sorbents were synthesized by impregnation of extruded activated carbon honeycombs and tested for the purification of simulated biogas from H_2_S at room temperature. Fresh, exhausted, and thermally regenerated honeycomb catalytic sorbents were characterized to shed light on the specific nature of S-species formed upon catalytic oxidation, the maximum capacity, and the efficacy of a simple thermal regeneration treatment at 600 °C [269].

Evolved gas analysis by mass spectrometry was fundamental to studying a fluid catalytic cracking unit that emits a large amount of flue gas, which is a major concern of environmental protection supervision. Wet flue gas desulfurization technologies have been widely used to control the emissions of SO_2_ in refineries, consequently, stack tests for pollutants emission of a typical FCC unit were conducted by Ju and by Song with respective coworkers. The emission characteristics indicated water vapor in the flue gas, which indirectly leads to large quantities of salt pollutants entrained in the flue gas. The EGA-MS analysis showed that nitrate sulfate could be decomposed and ammonia, SO_2_, and water vapor are released as reaction products in the form of gas [270,271].

A montmorillonite intercalated with Cu(II)-phenanthroline complex was prepared by Castellini et al. to capture gaseous H_2_S under mild conditions. The capture was studied over time and a mechanism of action was proposed by mass spectrometry evolved gas analysis [272].

## 23. Applications to Aluminium Industries

A large amount of spent carbon cathode block is produced after electrolytic pot failure. The spent cathode carbon block from aluminum reduction cells is considered to be a hazardous material due to containing a large number of soluble fluoride salts and toxic cyanides. Based on TG/DSC-MS system, Li and coworkers studied the characteristics of release and conversion of fluorides and cyanides during heat treatment of the spent cathode carbon block. The results indicate that the release of fluorides was steady. The cyanides were effectively decomposed at high temperatures and Ar–O_2_ atmosphere, with around three-quarters of the cyanides being converted to the N_2_ and nearly a quarter being to the NO. Finally, analysis of the flue gas composition indicated that it had a more complicated composition [273,274].

An unavoidable but reusable waste so as to enhance a more circular waste utilization is spent on potlining generated by the aluminum industry. The combustion mechanisms evolved gasses, and ash properties were characterized dynamically in response to elevated temperature and heating rates. Fluoride volatilization and combustion products were characterized by Sun et al. [275].

## 24. Applications to Explosives and Propellants

Thermal decomposition, kinetics parameters, and evolved gases during pyrolysis of energetic materials using different techniques have been widely studied experimentally and theoretically for a long time. The overall reaction processes and the conditions under which these can be achieved and the quality and quantity of evolved gases obtained during their thermal decomposition were collected in a specific review proposed by El-Sayed [276].

An innovative approach for the assessment of the real in-service-time and the equivalent in-service-time of double base rocket propellants is of utmost importance for the safe storage and use of weapon systems as well as the efficiency of accelerated aging plans. Four rocket propellants with similar chemical composition and different natural aging have been artificially aged by Chelouche and coworkers, and investigated by vacuum stability testing. The volume of the evolved gases was found to decrease with natural/artificial aging. The results showed excellent discrimination according to their stability thermal properties and the developed model provides more conceivable values, with asymptotic behavior against artificial aging duration evolution, thus overcoming some shortcomings of the common generalized van’t Hoff formula [277].

A new energetic coordination polymer composite, namely GO-Cu(II)-AmTZ, has been synthesized by 3-amino-1,2,4-triazole, and multifunctional graphene oxide coordination with Cu(II). Thermal analysis techniques including thermogravimetry coupled with mass spectrometry (TG/MS) were employed to determine the decomposition mechanisms, indicating its promising application as a combustion catalyst for improving the thermal-catalytic decomposition performance of energetic materials [278].

Izato and coworkers assessed the pyrolysis of a well-established gas generating agent consisting of a mixture of guanidine nitrate with basic copper nitrate, using thermoanalytical techniques in conjunction with high-resolution mass spectrometry (EGA-HRMS). This instrumentation simultaneously determined mass changes and heat flow and also permitted the evaluation of evolved gases on an accurate mass basis. The pyrolysis of a mixture of GN and BCN was proved to provide a synergistic effect that increases the heat and gas output [279].

Song and coworkers studied the properties of innovative energetic fibers prepared by the electrospinning method. In addition to spectroscopic characterizations, EGA-MS analysis proved the thermal decomposition products and the results of the energy performance evaluation showed that the standard specific impulse was remarkably higher than the state-of-the-art propellant. Therefore, these fibers are revealed as high-energy materials with very low sensitivity [280].

Thermodynamic and EGA-MS analyses were carried out to explore the thermal decomposition of an eutectic mixture by Kou et al. The results showed that the compatibility of the components of the eutectic is not perfect because of the intermolecular forces between the raw materials. The eutectic has the characteristics of high energy and insensitivity and is expected to replace TNT-based molten cast explosives [281].

Organic inner salt structures are ideal backbones for heat-resistant energetic materials and systematic studies of the thermal properties of energetic organic inner salt structures are crucial to their applications. Zhou and coworkers reported comparative thermal research of two energetic organic inner salts with different tetraazapentalene backbones. Detailed thermal decomposition behaviors and kinetics were investigated through thermoanalytical methods, showing that the thermal stability of the inner salts is higher than most of the traditional heat-resistant energetic materials. Further studies on the thermal decomposition mechanism were carried out through the analysis of the evolved gases by coupling both mass spectrometry and Fourier-transform infrared spectroscopy [282].

Aluminum powder is widely used in solid propellants to improve specific impulses. The dense alumina on the surface, however, can reduce reactivity and heat release of Al since alumina and titanium carbide reacts to generate gas and form pores. Wang et al. improved the reactivity and the heat release of the obtained highly reactive composite particle with a promising application in solid propellants [283].

Although the thermal decomposition of nitrocellulose, has been comprehensively examined, the effects of nitrogen content on the pyrolysis behavior have not been clearly elucidated. The macro- and micro-structures of nitrocellulose samples with varying nitrogen contents were qualitatively and quantitatively analyzed by Chai et al. The pyrolytic behavior was examined using EGA-FTIR-MS coupled analysis [284].

The desire to develop a benign burn rate modifier for propellants has accentuated polymeric carbon nitride as a potential candidate for the thermal decomposition of ammonium perchlorate. Mani et al. synthesized composites of leaf-shaped CuO and gCN via a facile sonochemical approach to determine the decomposition kinetics. From evolved gas analysis evolution of NO, Cl, HCl, N_2_O/CO_2_, NO_2,_ and Cl_2_ fragments were detected [285].

A systematic investigation of the effects of different copper catalysts on the thermal behavior of ammonium dinitramide was proposed by Li et al. Detailed information on evolved gas products on pyrolysis and oxidative degradations were obtained and revealed that copper chromite and β-Cu exhibit higher catalytic activity than copper chromate due to their greater acceleration of thermal decomposition [286].

The thermal decomposition and thermal reaction process of PTFE/Al/MoO_3_ fluorine-containing thermite at different temperatures were investigated by mass spectrometry coupling tests. The results showed that PTFE served as both oxidant and reductant in fluorine-containing thermite [287]. Hydroxylammonium nitrate monopropellant with its low sensitivity, volatility, and toxicity, not only promises a safer substitute for hydrazine but also offers a higher specific impulse and a positive oxygen balance. Agnihotri and Oommen primarily explored the decomposition mechanism and underlined kinetics for a newly developed cerium oxide-based catalyst. The reaction mechanism for both thermal and catalytic decomposition was proposed based on evolved gas analysis and the mechanism proposed was able to describe variable kinetic parameters extracted from the isoconversional method [288].

A high-energy cyclo-pentazolate salt with 90.98% nitrogen content, was synthesized by a simple metathesis reaction and characterized by ^15^N nuclear magnetic resonance, infrared and Raman spectroscopy, elemental analysis, EGA-MS, and differential scanning calorimetry. Results proposed LiN_5_ as an environmentally primary explosive [289].

The thermal decomposition of ammonium perchlorate-based molecular perovskite was studied by simultaneous coupling of FTIR-MS simultaneous evolved gas analysis [290].

The thermal decomposition behavior of heterogeneous mixtures of ammonium perchlorate with two organic additives (4, 4′-Bipyridine and biphenyl) was investigated and reported by Salehi and Eslami. The explosive decomposition behavior of prepared solid heterogeneous mixtures and the evolved gaseous products of decomposition were monitored. The change in the composition of volatile products indicates a change in the mechanism of ammonium perchlorate decomposition from proton transfer to electron transfer [291].

A state-of-the-art review focused on the thermal decomposition of energetic materials using EGA-FTIR and EGA-MS was reported by Benhammada and Trache [292].

## 25. Applications to Batteries and Conducting Materials

A TG-MS hyphenated characterization was proposed to analyze the thermal stability of cathode materials, including the thermal decomposition, the reaction thermal characteristics with the electrolyte, and the effects of different states of charge and electrolyte. For the lithium cobaltate cathode material, the reason why the O_2_ increased in Li_x_CoO_2_ was systematically explored. The results showed that the layered structure of the cathode materials is broken with the cut-off voltage increasing, resulting in the thermal stability decreasing, and even the thermal decomposition to release more oxygen with the increase of the amount of delithiation [293]. Nanostructured carbon-coated composite cathode materials were prepared by mechanochemically assisted solid-state synthesis, and the characterization, including evolved gas analysis, showed that the particle size and porosity strongly depend on the chosen precursors and intensity of mechanical impact [294].

Various nitrogen doping carbon materials, considered to be promising candidates for Na^+^ storage anodes, have been successfully controlled and synthesized by small-molecule polymerization methods. Their synthesis process has been verified by NMR, MALDI-TOF, TG-MS, and other techniques. When serving as sodium-ion battery anodes, these materials, dominated by a high concentration of pyrrolic N, exhibited a satisfactory reversible capacity [295].

Chemical solution deposition methods such as low-fluorine metal-organic decomposition, are nowadays widely employed in the production of superconducting films. The investigation of the chemical reactions that convert the organic precursors into the pyrolysis product is essential for the optimization of the pyrolysis step, and, ultimately, for the comprehension of the growth process. A detailed analysis of the single-salt precursors, of the ternary precursor solution and its thermal decomposition, was carried out by Piperno et al. to identify the precursors and propose a reaction path for pyrolysis [296].

Storage of nickel-rich LiNi_1–x–y_Mn_x_Co_y_O_2_ positive active materials under improper environmental conditions brings about undesirable surface reactions causing poor Li-ion cell electrochemical performances and gas generation. A detailed stepwise investigation of the increased reactivity of these active materials was reported by Martinez and coworkers. EGA-MS investigation allowed proposing the formation and the decomposition mechanisms by the evolution profile in the 50–500 °C region. Interestingly, the results also highlighted an in situ lithiated layered oxide material reformation reaction [297].

The in situ tracking of the pyrolysis of a binary molecular Zn cluster was presented with one brucite disk and two mononuclear fragments to porous carbon using TG-MS from 30 to 900 °C. Following up on the spilled gas product during the decomposed reaction of zinc cluster along the temperature rising, three key steps were observed [298].

Besides high energy density, fast-charging capability, and costs, the safety of Li-ion batteries is fundamentally important, even after long-term usage or abusive conditions. A new combination of accelerating rate calorimetry coupled with a mass spectrometer, as well as cell resistance and audio recording, was applied to study commercial 18650-type Li-ion cells. This novel setup allows following the electrochemical and thermal behavior simultaneously of the evolved gases during cell venting and thermal runaway [299].

Lithium borohydride, a well-known complex hydride with its high hydrogen capacity, has shown its application as a solid electrolyte for Li-ion batteries. It has been employed as a solid electrolyte with Bi_2_Te_3_ nanosheets as anode material for lithium-ion batteries. The careful investigation of the solvothermal synthesis reaction using EGA-MS suggested the destabilization of LiBH_4_ [300].

## 26. Applications to Waste Materials

Power generation without environmental impairment is a challenging task. Abedeen and coworkers studied the pyrolysis of discarded bus/truck tires with zeolite ZSM-5. A fixed-bed reactor was employed to pyrolyse scrap tires at a temperature range of 300–580 °C. Furthermore, it investigated the temperature and catalyst-to-tire ratio impact on the evolved oils’ generation and composition of hydrocarbons in the upgraded pyrolytic oils. The investigation identified a higher amount of o-xylene, toluene, naphthalene, and d-limonene in derived oil, denoting the capability of the evolved oil as an alternative energy source and value-added chemical substances [301].

EGA by TGA applied to plastics from waste electrical and electronic equipment showed a decrease in the total weight loss and the changes in weight loss stages, implying the thermal stabilities of the plastics drop [302].

In the context of waste upgrading of polyethylene terephthalate (PET) by pyrolysis, Dhahak and coworkers presented three on-line mass spectrometric techniques with soft ionization for monitoring the emitted decomposition products and their thermal dependent evolution profiles. Single-photon ionization (SPI at 118 nm/10.5 eV) and resonance-enhanced multiple photon ionization (REMPI at 266 nm) were used with time-of-flight mass spectrometry (TOF-MS) for evolved gas analysis (EGA-SPI/REMPI-TOFMS). Additionally, the chemical signature of the pyrolysis products was investigated by atmospheric pressure chemical ionization (APCI), ultra-high-resolution Fourier Transform ion cyclotron resonance mass spectrometry (FT-ICR MS) which enables the assignment of molecular sum formulas (EGA-APCI FT-ICR MS) [303].

As the global vehicle demand increases, the significance of waste materials generated from end-of-life-vehicles becomes more severe. A sustainable valorization platform for the vehicle wastes was suggested via pyrolysis process by Jung et al. As a case study, bumper waste was used as a feedstock material and multiple analytical tools were employed to identify and quantify the chemical constituents of the bumper waste. Because the bumper consists primarily of polypropylene, pyrolysis of the bumper produced hydrogen and different types of hydrocarbons and the product distribution was highly contingent on pyrolysis setups and operating conditions. When carbon dioxide was used as a reactive gas medium, additional CO production and suppression of coke formation were shown through gas-phase reactions. Therefore, it was assumed that CO_2_-assisted pyrolysis can be a considerable valorization platform for vehicle waste materials [304].

The activity of calcined scallop shell for the catalytic pyrolysis of wasted fishing net to the ε-caprolactam monomer was investigated in a micro-furnace type temperature programmable pyrolyzer, combined with a mass spectrometer. Evolved gas analysis indicated that the decomposition peak temperature in the absence of catalysts was around 420−480 °C, whereas the temperature decreased to 380−420 °C in the presence of calcined scallop shell or commercial CaO catalysts [305].

EGA-FTIR-MS was used to investigate the composition of waste cooking oil pyrolysis products evolved from a thermogravimetric analyzer. The results showed that the composition of gaseous products from waste cooking oil pyrolysis are diacylglycerols, monoglycerides, fatty acids, aliphatic aldehydes, aliphatic ketones, alkanes, alkenes, aromatic hydrocarbons, and CO_2_. Evidence is reported for the significant changes in the composition of gaseous products with the increase in temperature [306].

Co-combustion of dyeing sludge and rice husk is a promising energy-from-waste method. Wang and coworkers investigated and quantified the effect of rice husk additive on combustion performance, gas evolution (especially gaseous pollutants), and kinetics during sludge combustion by mass spectrometry evolved gas analysis. Results revealed that the introduction of rice husk improved the combustibility, burnout performance, and combustion stability of the sludge [307,308]. Zhang et al. showed that with the increase in the removal degree of alkali metal from rice straw, the release temperature of small molecules during the pyrolysis shifted to the high-temperature region, due to the catalytic effect of alkali metals on the escape of small molecules [309].

Synergism during the co-pyrolysis of microalgae, municipal solid waste, and their blends, was evaluated based on thermal decomposition pattern, evolved gases, heating rate and extent of thermal decomposition, and kinetic parameters. Three stages of devolatilization attributed to dehydration, devolatilization of major structural compounds of biomass, and decomposition of solid residues were noticed during the co-pyrolysis of biomass samples [310].

Zhu et al. investigated syngas production from petrochemical sludge and sawdust co-pyrolysis by EGA-MS and fixed bed reactor. The effects of pyrolysis temperature and interactions on gas evolution behavior, product distribution, and gas compositions were elucidated. Pyrolysis results showed that high temperature favored the gas production and there was a distinct increase in gas yield when the temperature exceeded 700 °C. The activation energy during the co-pyrolysis process was reduced due to the interaction [311]. Detailed investigations of the CuCl_2_ hydrolysis step of the Cu–Cl thermochemical cycle were carried out to obtain a performance evaluation of fixed bed hydrolysis [312].

Oil-based drill cuttings and waste polyvinyl chloride are both solid wastes. The gas, solid and liquid phase products of pyrolysis/co-pyrolysis were characterized and analyzed using thermal analysis, EGA-MS, GC-MS, and XRD. The gas product of pyrolysis/co-pyrolysis indicated the inhibitory effect on HCl emission due to the positive synergic effect [313]. A detailed study on the mechanism and kinetics of waste Polypropylene cracking oxidation by EGA-MS and in situ FTIR was reported by Xing et al. [314].

The characteristic parameters, evolved gases, reaction mechanisms, and ash conversions of the durian shell combustion were quantified by coupling thermal analysis with mass spectroscopy, Fourier transform infrared spectroscopy, and X-ray fluorescence spectra analysis, the main evolved gas being CO_2_, with no SO_2_ emission [315].

A cleaner chlorination thermal treatment for the recovery of gold from waste materials generated in gold manufacturing was reported by Li et al. A chlorination thermal treatment was conducted and EGA-MS analyses showed that Cl_2_ was the main compound participating in the chlorination reaction, and almost no HCl was generated [316].

## 27. Applications to Food

A novel method for the determination of oil boiling point distributions by thermogravimetric analysis coupled online with a mass spectrometer (TG-MS) was reported. The results obtained by this method differ less than 10% from those obtained by gas chromatography simulated distillation (GC-SIMDIS). The initial temperature of the cracking reaction when oil evaporates was determined by the online MS signal of small gas products [317].

Synchrotron radiation photoionization mass spectrometry (SR-PIMS) coupled system was utilized to online monitor the evolved gaseous compounds during the tea roasting process. By virtue of the “soft” ionization and fast data acquisition characteristics of SR-PIMS, dozens of aroma compounds including alcohols, aldehydes, furans, and nitrogen- and sulfur-containing species were detected and identified in real-time. The formation mechanisms of evolved compounds could be proposed according to the step-by-step formation process. The time-resolved results were demonstrated to be applicable in the evaluation of different roasting processes by statistical analysis [318].

Two studies published by Polat and Sayan and by Bejenari and coworkers aimed to investigate the pyrolysis characteristics and kinetics of spent coffee waste. The evolved gases detected during the decomposition were characterized by EGA-MS and primarily consisted of water, methane, and carbon dioxide [319,320].

EGA-MS and analytical pyrolysis coupled with GC/MS were used to characterize both the volatile and non-volatile fractions of six commercially available spices. As a new approach, EGA-MS was used to establish thermal degradation regions, and Py-GC/MS was used to obtain compositional information on each region separately using double-shot pyrolysis. This study demonstrates that EGA-MS and Py-GC/MS provide the same advantages of solid-phase microextraction and increase the range of detectable products by performing high-temperature desorption and degradation of the non-volatile fraction of spices. In addition, this approach provided both qualitative and semi-quantitative data [321].

A simple procedure was proposed for the analysis of Oenococcus oeni bacterium by Napoli et al. MS/MS data processing allowed for the identification of phosphorylation sites [322].

An innovative screening platform was proposed for the monitoring of the quality of milk using simultaneous multicomponent analysis. To optimize the platform, milk specimens with different origins and compositions were considered and prediction models were developed by chemometric analysis [323].

Sorbic acid and its potassium and calcium salts used as food preservatives and sorbic chloride were studied to characterize their melting and decomposition behavior in heating and cooling processes. Both potassium and calcium salts decomposed in temperatures higher than the acid without melting, producing the respective carbonates and oxides as final residues [324].

## 28. Applications to Space Materials

Evolved gas analysis has been successfully applied by Verchovsky and coworkers to the studies of meteorites and Apollo lunar samples. It consists of linear heating of a material with the registration of the released volatile compounds, typically using a spectrometric technique, to develop a Quantitative Evolved Gas Analysis (QEGA) technique using in-house custom-built Finesse mass spectrometry system. The method was tested using simple chemical compounds as references, such as CaCO_3_, which give well-known amounts of pure gases during their thermal decomposition [325,326].

The Sample Analysis at Mars (SAM) instrument on the Curiosity rover detected evidence of oxychlorine compounds (i.e., perchlorates and chlorates) in Gale crater, which has implications for past habitability, diagenesis, aqueous processes, interpretation of in situ organic analyses, understanding the martian chlorine cycle, and hazards and resources for future human exploration. Pure oxychlorines and mixtures of oxychlorines with Mars-analog phases have been analyzed for their oxygen and hydrogen chloride releases on SAM laboratory analog instruments in order to constrain which phases are present in Gale crater [327,328].

The 2019 Aguas Zarcas CM2 meteorite is the most significant carbonaceous chondrite CM2 fall since Murchison in 1969. Samples were collected immediately following the fall and studied by Garvie providing the rare opportunity to analyze the bulk mineralogy of a CM2 largely free of terrestrial contamination. The gases detected by EGA are dominated by water and CO_2_, largely derived from the dehydroxylation and decomposition of serpentine and calcite, respectively. In addition, gases are detected with masses matching SO_2_/S_2_ and H_2_S [329].

The Cumberland drill sample from the Sheepbed mudstone in Gale Crater, Mars, revealed the first evidence of an indigenous Martian organic molecule, chlorobenzene, with the Sample Analysis at Mars instrument on Curiosity. A mineralogical analog of the Cumberland sample was created to aid in the understanding of the precursor organic molecule(s) that led to the detection of chlorobenzene. The evolved gas analysis showed similarities with the Cumberland EGA on Mars in terms of the major volatiles. The quantification of chlorobenzene led to the prediction of organic precursor abundance on Mars of hundreds, if not thousands, of parts per million by weight [330].

Vera Rubin ridge is a topographic high within the layers of Mount Sharp, Gale crater, that exhibits a strong hematite spectral signature from orbit. Evolved gas analyses of Sample Analysis at Mars were performed on three samples from the ridge and one from directly beneath the ridge. SAM evolved H_2_O data suggested the presence of an Fe-rich dioctahedral smectite, such as nontronite, in the sample from beneath the ridge. Several volatile detections suggested trace-reduced sulfur sources, such as Fe sulfides and/or S-bearing organic compounds. HCl released from all samples likely resulted, in part, from trace chloride salts [331].

## 29. Applications to Pharmaceuticals

Betulin has become an exceedingly popular potential natural product, providing multiple pharmacological and biological activities, including anti-cancer, anti-viral, and anti-inflammatory benefits. The innovative application of a hyphenated system thermogravimetry–differential thermal analysis coupled with electron ionization or photoionization mass spectrometry was employed. The MS was equipped with the skimmer-type interface as a real-time and onsite analysis technique. Four solvatomorphs of Betulin were analyzed for the first time and five main volatile gaseous species were clearly identified [332].

Drospirenone is a fourth-generation progesterone that has been widely used in oral contraceptives for women because of its safety and few side effects in terms of pharmacological activity. A new solvatomorph (crystal form C) with dimethyl sulfoxide was identified and characterized for the first time through a TG-MS coupled system [333].

Chronothanatology has always been a challenge in forensic sciences. Therefore, the importance of a multidisciplinary approach for the characterization of matrices (organs, tissues, or fluids) that respond linearly to the postmortem interval is emerging increasingly. Risoluti et al. proposed a novel approach to the study of the vitreous humor since it is particularly suitable for studies aimed at assessing time-related modifications because it is topographically isolated and well-protected [334].

## 30. Applications to Glass

With the focus on thermal insulation, foamed glass prepared from the mixture of waste cathode ray tube panel glass, Mn_3_O_4,_ and carbon could become such a material, assuming that its production efficiency could be improved. In light of this, Hribar et al. engineered the transfer of the foaming process from inert to an air atmosphere, without drastically disturbing the primary mechanism of expansion. Foaming of carbon-containing mixtures in air atmosphere is normally a challenge due to premature oxidation of carbon by the oxygen from the air. By EGA-MS it was systematically investigated how the addition of water glass affects the process. Further, it proposed an explanation of how water glass protects the carbon and it showed that the addition allows for the process to be successfully performed in the air atmosphere [335].

## 31. Applications to Paint

The structure of the polymeric fraction in an oil painting is believed to be strongly connected to the stability of the paint layers over time, but its molecular characterization is extremely difficult given the complex composition of a vegetable oil-based polymer. Vannoni and coworkers reported the implementation of a methodological approach for the systematic mass spectrometric investigation of the molecular features of the products of oxidative degradation and cross-linking of oil paint layers upon curing. Evolved gas analysis techniques coupled to mass spectrometry were used to analyze the evolution of compounds produced over seven months of natural aging, from the volatile products to the macromolecular and cross-linked fractions. The aim was to improve the fundamental molecular understanding of the curing process of oil paints and to investigate the balance between oxidative degradation and cross-linking when specific binder-pigment combinations are in place. Data clearly showed crucial differences among paints with time, which are mostly related to the cross-linked fraction [336].

## 32. Conclusions

The selected cited references demonstrate the usefulness of OLTI-EGA to obtain simultaneous information, either qualitative or quantitative. EGA can be successfully applied to very different matrices, allowing a correct interpretation of the releasing/decomposition process. The hyphenated approach does not need a sample pretreatment, is solvent-less, is relatively fast and the MS fragments unequivocally give the correct characterization. The main limitation to be considered is in the sample amount needed, usually higher than 5 milligrams for a reproducible MS spectrum.

## Abbreviations

EGA	Evolved gas Analysis
TG	ThermoGravimetry
TGA	ThermoGravimetric Analysis
MS	Mass Spectrometry
QMS	Quadrupole Mass Spectrometry
FTIR	Fourier-Transform InfraRed spectroscopy
UV	UltraViolet spectroscopy
XRD	X-ray Diffraction
PMMA	Polymethylmethacrilate
DART	Direct analysis in real time
DFT	Density Functional Theory
DSC	Differential Scanning Calorimetry
DTA	Differential Thermal Analysis
ICP-OES	Inductively Coupled Plasma–Optical Emission Spectroscopy
MOF	metal Organic Framework
DRIFTs	Diffuse Reflectance Infrared Fourier Transform Spectroscopy
SEM	Scanning Electron Microscopy
HRTEM	High-Resolution Transmission Electron Microscopy
FID	Flame Ionization Detector
Py-GC/MS	Pyrolysis-GasChromatography/Mass Spectrometry
PCA	Principal Component Analysis
